# Small and Simple Molecular Structure Based Thermally Stable Ruthenium Precursor in Advancing Ruthenium ALD Process for Scaled Interconnect Metallization

**DOI:** 10.1002/advs.202519209

**Published:** 2025-11-23

**Authors:** Hideaki Nakatsubo, Debananda Mohapatra, Eun‐Soo Lee, Jeongha Kim, Iaan Cho, Masato Iseki, Toshiyuki Shigetomi, Ryosuke Harada, Sang‐Woong Na, Taehoon Cheon, Bonggeun Shong, Soo‐Hyun Kim

**Affiliations:** ^1^ Graduate School of Semiconductor Materials and Devices Engineering Ulsan National Institute of Science and Technology (UNIST) Ulsan 44919 Republic of Korea; ^2^ Chemical Materials Development Department TANAKA PRECIOUS METAL TECHNOLOGIES Co., Ltd. Tsukuba Ibaraki 3004247 Japan; ^3^ Department of Materials Science and Engineering Ulsan National Institute of Science and Technology (UNIST) Ulju‐gun Ulsan 44919 Republic of Korea; ^4^ Materials R&D Center TaeguTec LTD. Daegu 42936 Republic of Korea; ^5^ Department of Chemical Engineering Hongik University Mapo‐gu Seoul 04066 Republic of Korea; ^6^ Department of Electrical and Electronic Engineering Yonsei University Seoul 03722 Republic of Korea; ^7^ R&D Center CTH Corporation Daegu 43018 Republic of Korea

**Keywords:** advanced interconnects, bulk‐like resistivity, high growth per cycle (GPC), high thermal stability, novel Ru precursor, Ru atomic layer deposition (ALD‐Ru), selectivity

## Abstract

Ruthenium (Ru) via atomic layer deposition (ALD) has emerged as a promising alternative to copper‐interconnects. For the first time, a small yet simple molecular structure Ru precursor, [Ru(trimethylenemethane (TMM))(*p*‐cymene)], with excellent thermal stability up to 400 °C is introduced that enables a high‐temperature ALD‐Ru process with a high growth per cycle of ≈1.28 Å cycle^−1^ and a short incubation period (≈8 cycles) on TiN, facilitating uniform, dense film growth. The process achieves low impurity levels and resistivities as low as 10.6 µΩ cm at 350 °C without postannealing, approaching bulk Ru values (7.4 µΩ cm). Additionally, no Ru nucleation is observed on SiO_2_ even after 1000 cycles, indicating excellent substrate selectivity. Computational analyses confirm the substrate‐selective adsorption behavior of the precursor, favoring TMM‐terminated configurations on Ru and RuO_2_, while nucleation on SiO_2_ can be delayed. Fragmentation energy calculations further support the precursor's thermal robustness through strong Ru─ligand bonding. Advanced crystallography/microstructure analysis using electron backscatter diffraction reveals that the enhanced grain growth and the formation of low‐energy coincidence site lattice boundaries are critical for minimizing resistivity, which is supported by combined Fuchs–Sondheimer–Mayadas–Shatzkes modeling. These findings position the new Ru precursor as a robust candidate for durable, scalable ALD‐Ru processes in advanced interconnect technology.

## Graphical abstract

A thermally robust (≈400 °C), critically small‐size‐simple ligand structure ruthenium (Ru) precursor enables exceptional growth per cycle (≈1.28 Å cycle−1), short incubation (≈8 cycles), ultralow resistivity (8.65 µΩ cm) and outstanding substrate selectivity via atomic layer deposition (ALD) process at high temperatures, overcoming prior Ru‐ALD limitations, offering a scalable, impurity free pathway for next generation interconnects beyond copper.

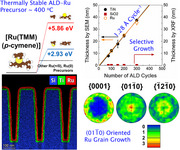

## 1 Introduction

In the last few decades, continued downscaling of integrated circuits (ICs) following Moore's law has significantly increased interconnect density.^[^ ^1^, ^2^ ^]^ This drives the need for advanced interconnect miniaturization and requires novel materials to mitigate parasitic effects and RC delays on semiconductor devices at the nanoscale. Copper (Cu) has been widely used for wiring in the semiconductor field since the late 1990s. The challenging long electron mean free path for Cu, compared to ruthenium (Ru), causes severe deterioration of the semiconductor device functionality when the dimension is scaled down to below a few nanometers. With the demand for more Moore and continuous downscaling of ICs, the channel material must be shrunk under the channel length, limiting Cu metallization.^[^ ^3^, ^4^ ^]^ This is primarily due to the drastic increase of Cu's resistivity arising from the scattered electrons at the surface and grain boundaries when the semiconductor device dimensions are smaller than Cu's electron mean free paths, i.e., ≈40 nm.^[^ ^5^, ^6^, ^7^, ^8^, ^9^ ^]^ Moreover, Cu itself is susceptible to other reliability concerns, including electromigration (EM),^[^ ^10^, ^11^ ^]^ and time‐dependent dielectric breakdown,^[^ ^12^, ^13^ ^]^ due to its inherent chemical and thermal characteristics. These issues are aggravated by continued scaling via wiring miniaturizations.^[^ ^14^ ^]^

To mitigate these fabrication challenges, diffusion barrier layers, such as tantalum nitride (TaN), have been employed to prevent Cu from migrating into surrounding dielectrics.^[^ ^15^ ^]^ However, the barrier layer's thickness at the nanoscale has become a major limiting factor in terms of both resistivity and reliability in further miniaturization of wiring.^[^ ^16^ ^]^ As a result, researchers have investigated alternative interconnect materials to replace Cu. Theoretical studies suggest that certain platinum group metals [e.g., Ru, rhodium (Rh), iridium (Ir)] and transition metals (e.g., cobalt, molybdenum) may offer to promise interconnect alternatives due to their relatively short electron mean free paths, low bulk resistivity, and better reliability by virtue of high melting point.^[^ ^6^, ^17^ ^]^ Among potential interconnect materials, Ru is particularly promising due to its low resistivity with short electron mean free paths, high melting point, and chemical stability as a noble metal. It has high EM resistance inherently and does not require a diffusion barrier layer, making it suitable for advanced interconnect applications to overcome the miniaturization challenges. Ru also has economically attractive features compared to Rh and Ir noble metals in terms of material cost. Ru atomic layer deposition (ALD‐Ru) provides distinct advantages, such as excellent step coverage in high‐aspect‐ratio structures and precise control over film thickness at the atomic scale due to its self‐limiting surface reactions compared to those of physical vapor deposition and chemical vapor deposition (CVD). Importantly, ALD can yield high‐purity films with lower resistivity than conventional CVD and other thin film deposition methods.^[^ ^18^, ^19^, ^20^ ^]^

Single metal ALD‐Ru process with O_2_ as a coreactant is well established in the literature and summarized with their various growth kinetics and thin film properties in Table S1 (Supporting Information). To be the specific connection to our current work on novel Ru precursor design, the previously reported high‐valence Ru precursors, such as [Ru(thd)_3_] and [Ru(EtCp)_2_], often suffer from low growth per cycle (GPC) 0.36 Å cycle^−1^ and extended incubation periods over ≈250 cycles^[^ ^21^ ^]^ and 0.49 Å cycle^−1^ over ≈200 cycles,^[^ ^22^, ^23^ ^]^ respectively, posing practical limitations. By contrast, zero‐valent Ru precursors like IMBCHDRu and EBCHDRu demonstrate higher GPCs and shorter incubation periods (0.89 Å cycle^−1^ in 11 cycles^[^ ^24^, ^25^ ^]^ and 1.0 Å cycle^−1^ in 2 cycles,^[^ ^26^ ^]^ respectively). However, these films exhibit relatively high resistivity, ≈30 µΩ cm for IMBCHDRu^[^ ^25^ ^]^ and ≈20 µΩ cm for EBCHDRu,^[^ ^27^ ^]^ significantly above the bulk Ru resistivity of 7.4 µΩ cm. This performance gap is attributed mainly to poor thermal stability (typically <300 °C^[^ ^24^, ^26^, ^27^ ^]^), which limits process temperatures, because high temperature increases unwanted impurity incorporation and makes the deposition process uncontrolled with unusual GPC due to precursor self‐decomposition.^[^ ^24^, ^28^ ^]^

Meanwhile, a high process temperature provides a lower resistivity of Ru thin film, attributed to the large and well‐developed polycrystalline grain size.^[^ ^26^ ^]^ Both high GPC with self‐limiting behavior and better thin film properties are critical for the desired ALD‐Ru process. Hence, the critical size and ligand structure of the Ru precursor molecule need to be optimized in terms of their ligand simplicity, not only to increase the adsorption density of those nonoxidative precursor molecules and to decrease a ratio of non‐Ru elements per precursor unit which leads to film impurities, but also to obtain their chemical persistence not to proceed self‐decomposition at higher temperatures. When no oxygen atoms are directly linked or bonded to the ruthenium metal atom centered in the Ru precursor, the possibility of achieving high‐purity metallic Ru thin films is high. Nonoxidative precursors avoid oxygen incorporation into the pristine films as contaminants along with carbon or nitrogen. Moreover, it will also provide a chemically stable adsorbed species, resulting in an optimal ALD temperature window with a feasible growth rate due to the lack of oxygen containing ligand because precursors having such ligands (e.g., β‐diketonates, monocarbonyl) easily lose its stability and volatility through an irreversible break of coordination,^[^ ^29^ ^]^ for example, ligand conversion into light gaseous molecules^[^ ^30^ ^]^ or ligand dissipation into the gas phase.^[^ ^31^ ^]^ Therefore, to overcome these challenges, introducing a small yet simple molecular structure based novel Ru precursor without involving any oxygen in the structure with high thermal stability is imperative to withstand the high‐temperature ALD‐Ru process. This precursor achieves a high GPC (≈1.28 Å cycle^−1^) and a short incubation period (≈8 cycles) with thermal stability up to 400 °C. Crystallographic analysis via electron backscatter diffraction (EBSD), next‐generation aberration‐corrected ultrahigh‐resolution transmission electron microscope (UHR‐TEM), secondary ion mass spectrometry, and advanced X‐ray diffraction and modeling based on the Fuchs–Sondheimer and Mayadas–Shatzkes (FS–MS) approach revealed that the grain growth and the formation of low‐energy grain boundaries, such as coincidence site lattice (CSL) boundaries, are critical to resistivity reduction approaching bulk Ru resistivity values. These microstructural improvised features are effectively promoted by high‐temperature ALD with excellent substrate selectivity, made possible by the high thermal stability of the new precursor. This unique combination of high growth rate, low resistivity, thermal robustness, and substrate selectivity positions this novel Ru precursor as a highly promising candidate for a high‐performance ALD‐Ru process for next‐generation interconnect semiconductor technology.

## 2 Results and Discussion

### 2.1 Ru Precursor Chemical Structure and Thermal Properties

Figure S1A (Supporting Information) shows the chemical structure of [Ru(TMM)(*p*‐cymene)]. The structure is similar to a previously reported Ru precursor tricarbonyl(trimethylenemethane)ruthenium [Ru(TMM)(CO)_3_],^[^ ^28^ ^]^ having trimethylenemethane (TMM) ligand; however, this new precursor has only a hydrocarbon ligand in the molecule, having isopropylmethylbenzene (*p*‐cymene) instead of monocarbonyl. Figure S1B (Supporting Information) shows the TG curve of the precursor from ambient to 500 °C. The precursor starts to vaporize at around 100 °C and almost completely vaporizes at around 200 °C, proving its suitability for clean, impurity‐free thin film depositions at elevated temperatures. Practically no residue (less than 1%) after fully vaporizing indicates good vaporizing volatility characteristics and reactivity enhancement and is suitable for the ALD precursor. Next, the thermal stability of the precursor was evaluated by only supplying [Ru(TMM)(*p*‐cymene)] without any reactants for 10 min up to 400 °C to check whether the precursor was thermally decomposed spontaneously (refer to Section S1.1 in the Supporting Information for details). As shown in Figure S2 (Supporting Information), there is no apparent Ru signal from X‐ray fluorescence (XRF), indicating this precursor has excellent thermal stability up to 400 °C. Compared to other Ru precursors which have similar structures, for example, [Ru(TMM)(CO)_3_], having TMM ligand is thermally decomposed at 275 °C,^[^ ^28^ ^]^ and IMBCHDRu, having *p*‐cymene ligand is thermally decomposed at 310 °C.^[^ ^24^ ^]^ This means both TMM and *p*‐cymene ligands and their combination play a vital role in thermal stability, which is further discussed in Section 2.3.

### 2.2 Thin Film Growth Kinetics, Surface Self‐Limiting Characteristics, Selectivity, and Temperature Window of ALD‐Ru Process

**Figure 1:**
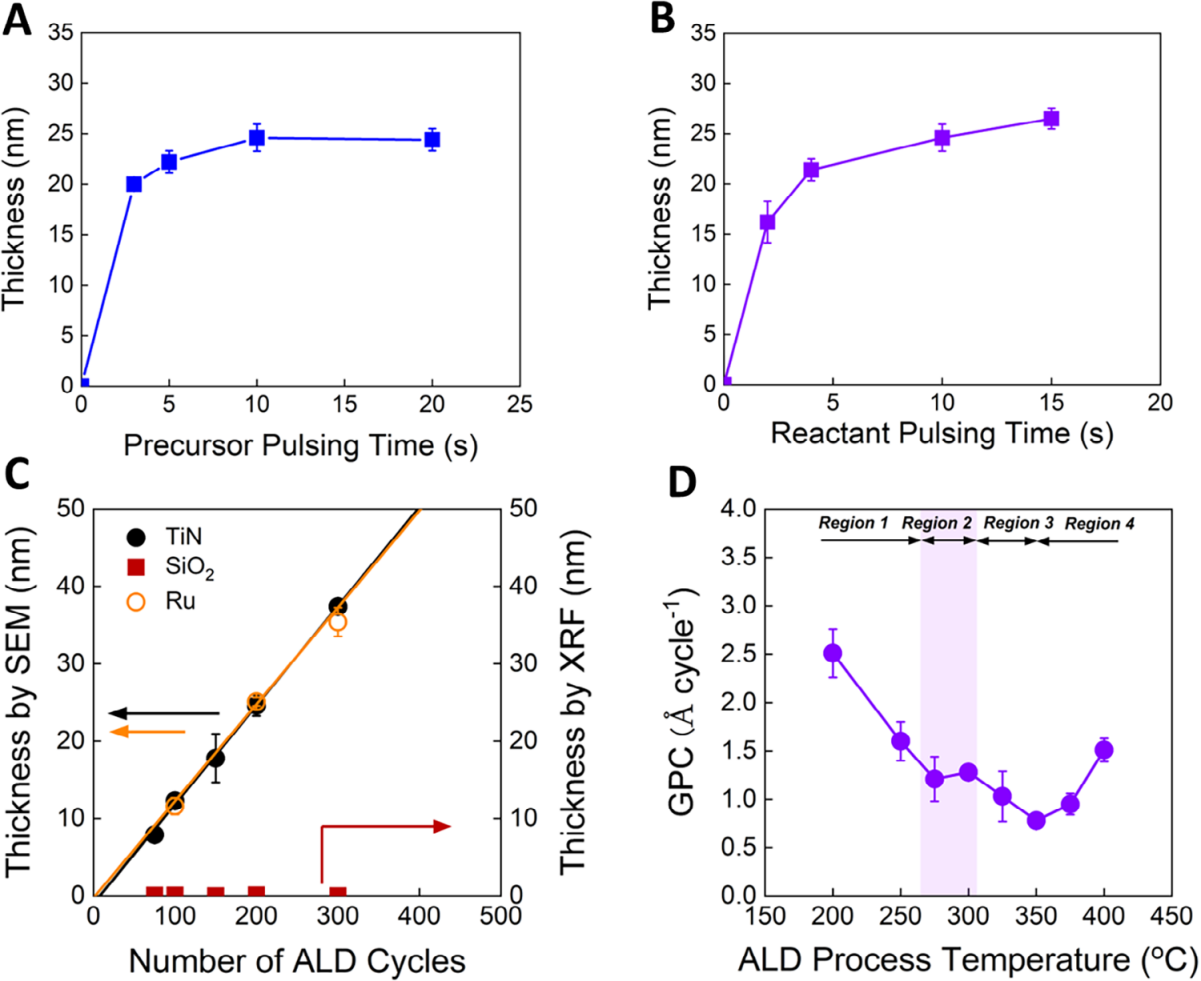
A) The thickness of ALD‐Ru film with precursor pulsing time, B) O_2_ reactant pulsing time at 300 °C on TiN substrate. C) The thickness of the ALD‐Ru thin films deposited under optimized pulsing conditions as a function of the number of ALD reaction cycles on TiN, SiO_2_, and Ru substrates. D) GPCs of the ALD‐Ru thin films deposited on TiN substrates at various ALD process temperatures under optimized pulsing conditions.

At first, we evaluated reactivity among various reactants and [Ru(TMM)(*p*‐cymene)] at 400 °C on TiN. O_2_ was used in this process as a reactant (refer to Section S1.1 in the Supporting Information for details), because other reactants such as H_2_, NH_3_, and H_2_O did not react well with [Ru(TMM)(*p*‐cymene)] (Figure S3, Supporting Information). It indicates that a reactant needs high reactivity because the ligands of the precursor might be chemically stable and strongly bond to the centered Ru atom. This is also reasonable in terms of the higher thermal stability of the precursor, as discussed in Section 2.1 above. Although nonoxidative reactants such as H_2_ and NH_3_ molecules can be attractive due to the advantage for oxidation‐free process, specific processes such as plasma‐enhanced ALD^[^ ^32^ ^]^ or high pressure ALD^[^ ^33^, ^34^ ^]^ might be required to improve the reactivity of reactants when those reactants are applied. Herein, we used O_2_ as a reactant to ensure sufficient reactivity and conducted further ALD‐Ru experiments including self‐limiting growth behavior, eliminating the possible growth by thermal decomposition of the Ru precursor. **Figure** **1**A shows the deposited Ru layer thickness on TiN evaluated by FE‐SEM with 200 cycles at 300 °C as a function of the Ru precursor pulsing time. The reactant pulsing time was fixed at 10 s, and each purging (precursor purging and reactant purging) time was also fixed at 10 s. The thickness increased up to 5 s, and saturated from 10 s, indicating typical self‐limiting growth characteristics for the model ALD‐Ru overall process. It demonstrates that the new precursor [Ru(TMM)(*p*‐cymene)] can be applicable to the ALD‐Ru process, showing high thermal stability that excludes the uncontrolled growth that could arise from thermal decomposition. The deposited Ru layer thickness as a function of the reactant (O_2_) pulsing time is also shown in Figure 1B. The precursor pulsing time and each purging were fixed at 10 s. The thickness was drastically increased to 5 s, then slightly increased up to 15 s. Although a slight increase was still observed at 15 s, the change was minimal, indicating that sub‐self‐limiting behavior to the reactant was observed. It can be interpreted that oxygen molecules can dissociate and adsorb on Ru surface and some oxygen atoms are partially incorporated into the most upper Ru surface layers and promote Ru deposition, similar behavior in Ru O_2_ ALD using ECPR at 255 °C.^[^ ^35^ ^]^ Böttcher and Niehus^[^ ^36^ ^]^ investigated the formation of subsurface oxygen in Ru metal below 800 K, revealing that oxygen atoms can diffuse into the Ru subsurface above 550 K in the article. Moreover, many previous reports related to Ru ALD using O_2_ reactant provided experimental evidence of a reaction between absorbate O species and Ru precursor during the precursor pulsing step, especially when adopting long O_2_ pulsing time and relatively high temperature.^[^ ^21^, ^37^, ^38^, ^39^, ^40^ ^]^ Therefore, we assume that the slight increase in Figure 1B is due to the reaction between the precursor and oxygen atoms, which adsorb on the surface and/or diffuse into the Ru subsurface, during the precursor pulsing step rather than the precursor's thermal decomposition.

Figure 1C shows the deposited Ru film thickness as a function of the number of ALD cycles on Ru, TiN, and SiO_2_ substrates at 300 °C. On Ru and TiN, the thickness increased linearly with the number of cycles, and their growth characteristics are almost the same. The GPC on TiN is 1.28 Å cycle^−1^, which is quite higher than the previously reported values of Ru precursors (Table S1, Supporting Information). The incubation period on TiN was also ≈8 cycles, showing great kinetic characteristics like zero‐valent Ru precursors. Interestingly, on the other hand, almost no Ru was deposited on SiO_2_. It indicates Ru growth was affected by the type of substrate, suggesting the possibility of selective deposition. Figure S4 (Supporting Information) shows the selectivity between TiN and SiO_2_ substrates up to 1000 cycles. The selectivity was evaluated using Equation (1),^[^ ^41^ ^]^ and XRF quantified the amount of deposited Ru on both substrates (Table S2, Supporting Information)

(1) $$\frac{\theta_{\mathrm{GA}}-\theta_{\mathrm{NGA}}}{\theta_{\mathrm{GA}}+\theta_{\mathrm{NGA}}}\approx \frac{t_{\mathrm{GA}}-t_{\mathrm{NGA}}}{t_{\mathrm{GA}}+t_{\mathrm{NGA}}}$$

(*θ*: coverage, *t*: layer thickness, GA: growth area, NGA: nongrowth area)

The selectivity was maintained as 100% even up to 1000 cycles, showing that excellent selectivity was confirmed. This selectivity performance is far beyond compared to other previously reported metal on metal area‐selective deposition (ASD) of Ru.^[^ ^42^, ^43^, ^44^, ^45^, ^46^ ^]^ A recent report providing Ru‐ASD at high temperatures mentioned the selectivity exhibition needs a high temperature to desorb the precursor on the nongrowth surface.^[^ ^47^ ^]^ One reason for this high selectivity might be the high process temperature, such as 300 °C. Figure 1D shows the GPC on TiN with the process temperatures from 200 to 400 °C. Four temperature regions with different growth kinetics might be considered. The first region is below 250 °C. In this region, higher GPC was observed, suggesting condensation of the precursor. The second region is from 275 °C to less than 325 °C, and GPC remains almost constant, indicating the so‐called ALD window. The third region starts from 325 °C, and GPC drastically decreases up to 350 °C, suggesting precursor desorption occurs. The fourth region is above 350 °C, and GPC increases with the process temperature despite its high thermal stability where the trend in fourth region is generally interpreted as the thermal decomposition of the precursor. One possible explanation is the contribution of subsurface oxygen. As we discussed in this section, oxygen can diffuse into the Ru subsurface at high temperatures. Additionally, the amount of oxygen content was drastically increasing above 600 K (≈2 ML at ≈600 K, and ≈9 ML at ≈700 K).^[^ ^36^ ^]^ Therefore, the increased trend of GPC can be interpreted as the increase of reacted oxygen during the Ru precursor pulsing step, even though the precursor desorption occurs at >325 °C. These results and interpretations may give insight into understanding Ru O_2_ ALD at high temperatures.

**Figure 2:**
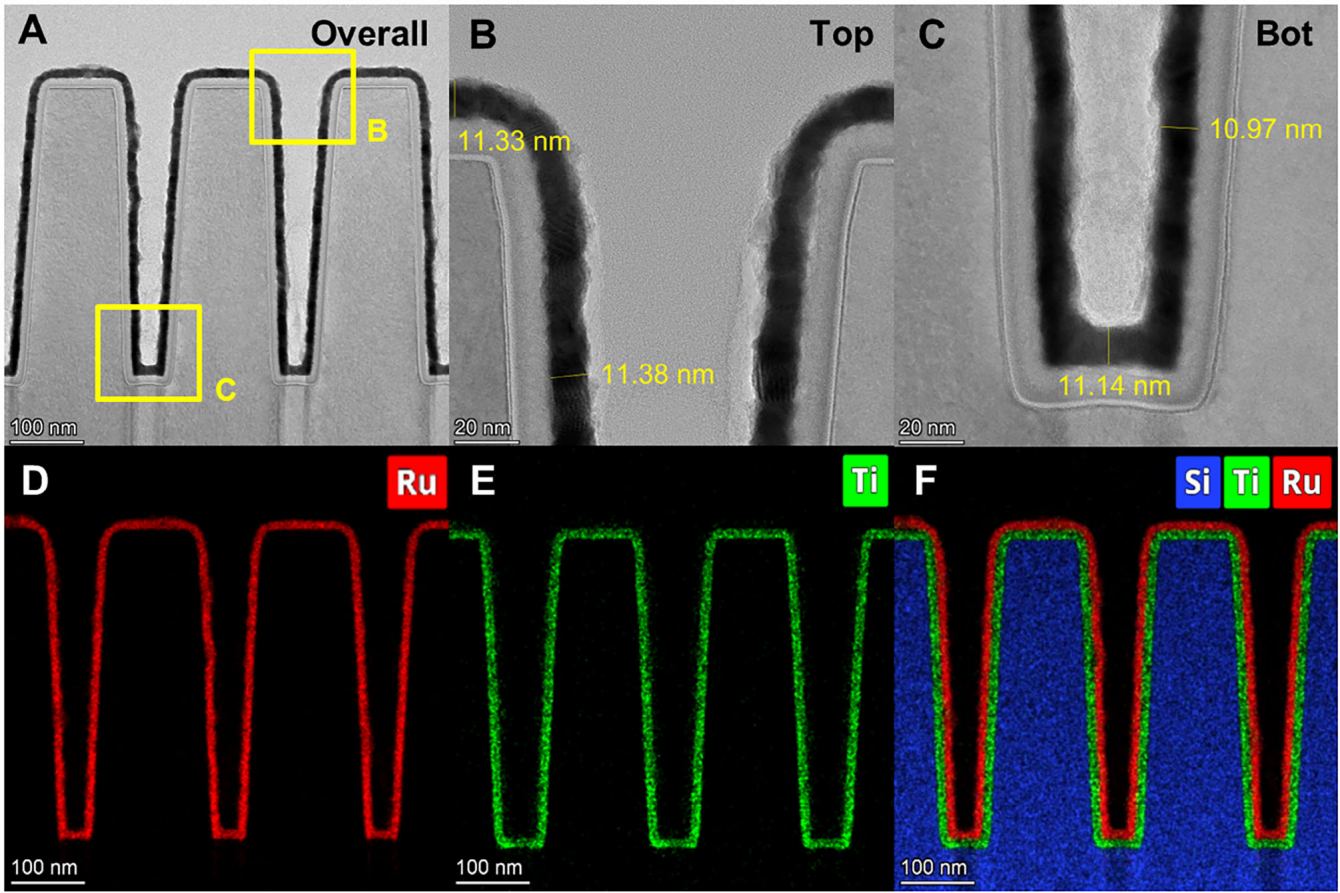
Advanced aberration‐corrected microstructure analysis, step coverage, and elemental confirmation of ALD‐Ru thin films. Cross‐sectional view transmission electron microscopy (XTEM) of A) overall, B) top of the trench, and C) bottom of the trench. Elemental mapping quantification for D) Ru, E) Ti, F) full Ru, Ti, and Si.

**Figure** **2**A–F shows the images of cross‐sectional view transmission electron microscopy (XTEM) and their elemental mappings with around 10 nm Ru film on TiN‐deposited trench with an aspect ratio of ≈4 (top width: ≈115 nm, bottom width: 65 nm, trench depth: ≈415 nm) by conducting [Ru(TMM)(*p*‐cymene)] O_2_ ALD process at 300 °C. Continuous Ru film was successfully deposited on TiN (Figure 2D–F). A high‐resolution image also revealed that deposited Ru atoms were well ordered along the growth direction (Figure S5, Supporting Information). Excellent step coverage >95% was confirmed with uniform and conformal Ru thin film (Figure 2B,C), indicating [Ru(TMM)(*p*‐cymene)] is applicable and promising for the fabrication related to nanoscaled Ru interconnects metallization, replacing Cu interconnects. A bottom‐up process for a high aspect ratio structure is also promising when using this new Ru precursor because of its outstanding selectivity.

### 2.3 Computational Evaluation on Adsorption Selectivity and Thermal Stability of [Ru(TMM)(*p*‐cymene)]

**Figure 3:**
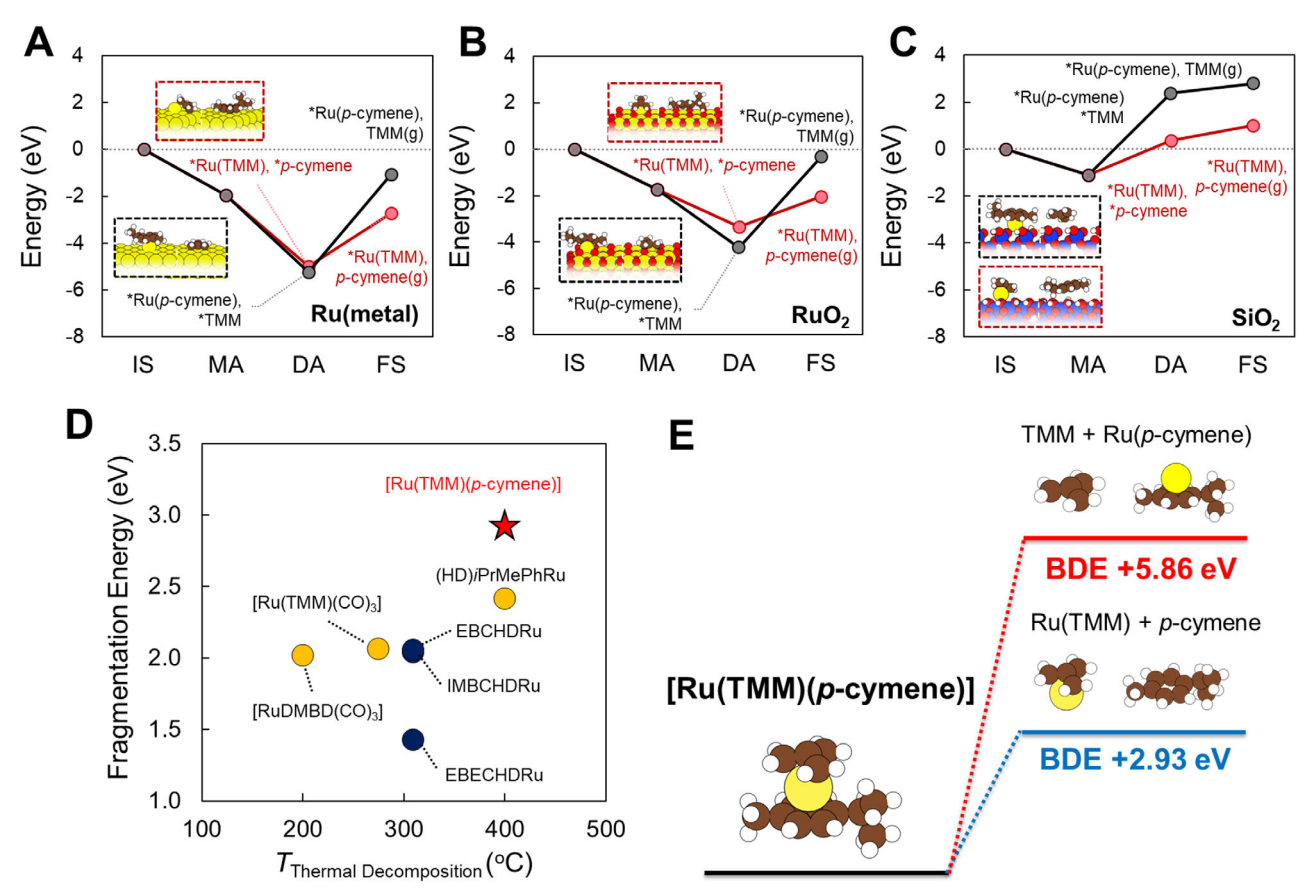
Adsorption and thermal stability analyses of the [Ru(TMM)(*p*‐cymene)] precursor. MLIP‐calculated adsorption energy diagrams on A) Ru, B) RuO_2_, and C) SiO_2_. Optimized structures at the dissociative adsorption (DA) step are included. Atom colors: Ru (yellow), C (brown), H (white), O (red), Si (blue). D) Correlation between experimental decomposition temperatures and DFT‐calculated fragmentation energies of various Ru(0) and Ru(+/0) precursors (homolytic and rearranged bond dissociation energies (BDEs) in yellow/red and navy, respectively; [Ru(TMM)(*p*‐cymene)] marked by a red star). E) Schematic of two possible dissociation pathways for [Ru(TMM)(*p*‐cymene)].

To investigate the substrate‐dependent adsorption mechanism of [Ru(TMM)(*p*‐cymene)], MLIP‐based energy profiles were calculated for Ru and RuO_2_, reflecting interfacial oxidation, and hydroxylated SiO_2_ surfaces (refer to Section S1.3 in the Supporting Information for details). Reaction energies for each mechanistic step, including the initial state (IS), molecular adsorption (MA), dissociative adsorption (DA), and final state (FS, involving ligand elimination), were determined based on Hess' law using total energies of the relevant configurations, as shown in **Figure** **3**A–C.

On both Ru and RuO_2_ surfaces (Figure 3A,B), DA involving cleavage of either the TMM or *p*‐cymene ligand was found to be exothermic, and comparable in magnitude, with only marginal additional stabilization observed on Ru. These results demonstrate that the precursor can be efficiently adsorbed and dissociated on both metallic and oxidized Ru surfaces. By contrast, adsorption on SiO_2_ (Figure 3C) was endothermic, particularly for TMM dissociation, reflecting a thermodynamic instability and pronounced substrate selectivity.

The endothermic energy to proceed from DA to FS corresponds to the ligand desorption energy. The desorption energy of *p*‐cymene was calculated as 2.27 eV on Ru and 1.31 eV on RuO_2_, whereas that of TMM entailed significantly higher values as 4.18 and 3.90 eV, respectively. These findings confirm that *p*‐cymene elimination is thermodynamically preferred on both surfaces, favoring the formation of surface‐bound *Ru(TMM) species under ALD conditions, which is consistent with the lower energy of the *Ru(TMM) at FS by over 1.7 eV on all surfaces. On SiO_2_, both ligands exhibited desorption energies below 1 eV, suggesting facile ligand removal irrespective of identity. DFT calculations were performed to validate the desorption energy of *p*‐cymene, and the MLIP and DFT results showed consistent trends (Figure S6, Supporting Information). Consistency with DFT was also confirmed for representative bulk and surface properties of Ru and RuO_2_, and for the internal bond lengths of the [Ru(TMM)(*p*‐cymene)] precursor (Table S3, Figure S7, Supporting Information).

To further evaluate the desorption kinetics, the ligands' surface lifetimes (*τ*) were estimated from desorption energies using first‐order Arrhenius kinetics (Table S4, Supporting Information). The *τ* of *p*‐cymene on Ru was over 10⁸ times longer than on RuO_2_, consistent with its more persistent adsorption on metallic surfaces. Additionally, the *τ* of TMM was higher than that of *p*‐cymene by more than 10^16^ times on Ru and 10^22^ times on RuO_2_, confirming its exceptionally strong surface retention across both substrates. These results imply that partial oxidation of the Ru surface during the initial ALD cycles promotes selective *p*‐cymene removal, stabilizing TMM‐terminated configurations and enhancing self‐limiting growth, leading to a high GPC as 1.28 Å cycle^−1^ due to the small TMM ligand with less steric hindrance. As surface oxygen is consumed through combustion reactions^[^ ^48^, ^49^, ^50^ ^]^ with hydrocarbon ligands, forming oxidized carbon and hydrogen species, the metallic Ru surface is gradually exposed, increasing the likelihood of coadsorption of TMM and *p*‐cymene and resulting in a slightly lower GPC than that of [Ru(TMM)(CO)_3_] (≈1.7 Å cycle^−1^). These findings highlight the dynamic influence of the surface oxidation state on precursor adsorption and reaction pathways during Ru ALD.

To assess the intrinsic thermal stability of the [Ru(TMM)(*p*‐cymene)] precursor and to compare it with alternative Ru(0) or Ru(+/0) precursors, DFT‐based fragmentation energy calculations were conducted. Eight different ligands as seen in Figure S8 (Supporting Information), namely CO, DMBD, EB, ECHD, CHD, HD, TMM, and *p*‐cymene, were incorporated into the precursor structures based on experimentally reported compounds. For each precursor, either the homolytic or rearranged bond dissociation was considered based on ligand structure, and the lower‐energy value was selected as the representative fragmentation energy (Figure 3D; Table S5, Supporting Information). For precursors containing CHD‐type ligands (e.g., EBCHDRu, IMBCHDRu, and EBECHDRu), intramolecular hydrogen transfer from the ligand to the Ru center enabled rearrangement into benzene‐type fragments,^[^ ^51^, ^52^ ^]^ yielding lower fragmentation energies than the homolytic route. By contrast, precursors with CO, DMBD, TMM, and *p*‐cymene ligands do not possess structurally labile hydrogens or substituent patterns that facilitate rearrangement, rendering homolytic dissociation a more appropriate representation of their thermal degradation pathway.

Under this framework, [Ru(TMM)(*p*‐cymene)] exhibited the highest fragmentation energy of 2.93 eV. As illustrated in Figure 3E, the dissociation energy for TMM was approximately twice that of *p*‐cymene, the latter of which was already highly endothermic. This result indicates strong coordination of both ligands to the Ru center, reflecting the notable thermal stability of the complex. A comparative evaluation with [Ru(TMM)(CO)_3_] further demonstrated that CO dissociation (2.06 eV) occurred with substantially lower energetic cost than *p*‐cymene removal, suggesting that *p*‐cymene plays a more significant role in enhancing the thermal robustness of the precursor. This interpretation is consistent with the experimentally observed stability of [Ru(TMM)(*p*‐cymene)] up to 400 °C.

### 2.4 Resistivity, Crystallographic Evolution, and Associated Properties of Deposited Ru‐ALD Thin Films

Figure S9A (Supporting Information) shows the resistivity of Ru on TiN as a function of deposited Ru film thickness at 300 °C. The sheet resistance of deposited Ru film was calculated by subtracting the sheet resistance of the underlayer TiN while assuming a parallel connection. Resistivity decreased with the layer growth, reaching 14.2 µΩ cm at ≈25 nm. This value is comparatively low compared to previously reported Ru precursors around the same thickness shown in Table S1 (Supporting Information) (for example, [Ru(TMM)(CO)_3_]: >20 µΩ cm ≈30 nm,^[^ ^28^ ^]^ IMBCHRu: ≈30 µΩ cm ≈20 nm^[^ ^24^ ^]^). Even in a thin region, it still shows a relatively small value of 34.9 µΩ cm at 4.1 nm. This indicates that this precursor is promising for Ru‐interconnects, especially in the sub‐10 nm region. A comparison of resistivity to other metals, such as Cu and Mo (Figure S9B, Supporting Information), Ru film resistivity of this work is better than Mo deposited by ALD at 400 °C,^[^ ^53^ ^]^ especially at less than 10 nm. In the case of Cu, deposited Cu on TaN with annealing (420 °C, 20 min) reported by Dutta et al.^[^ ^54^ ^]^ shows low resistivity at thick region (>10 nm). However, considering the thickness of the diffusion barrier for practical interconnects via filling, such as 3 nm TaN, the line width of Cu needs to be shortened by the thickness of both sides of the diffusion barrier (6 nm). Thus, Ru film resistivity of this work is superior to Cu at less than 10 nm because Ru does not need any diffusion barriers. It demonstrates that [Ru(TMM)(*p*‐cymene)] is one of the promising Ru precursors for replacing Cu‐interconnects. Figure S10 (Supporting Information) shows the resistivity with similar Ru thickness ≈30 nm on TiN as a function of the process temperature. The resistivity is quite high at 200 °C, exceeding 100 µΩ cm. At 250 °C, where many examples of zero‐valent Ru precursors are implemented, the resistivity is 22.0 µΩ cm, close to the previously reported value such as EBCHDRu at ≈20 µΩ cm^[^ ^26^ ^]^ at 225 °C and IMBCHDRu at ≈30 µΩ cm at 270 °C.^[^ ^24^ ^]^ Above 300 °C, which leads to resistivity degradation via thermal decomposition in some other Ru precursors,^[^ ^24^, ^28^ ^]^ the resistivity using this precursor gradually decreased with the process temperature and reached 12.3 µΩ cm at 375 °C. This is one of the lowest values in previously reported Ru‐ALD processes (Table S1 (Supporting Information), 13–36 µΩ cm). It indicates [Ru(TMM)(*p*‐cymene)] can exhibit high‐quality films by fully utilizing the advantages of high process temperature without thermal decomposition. At 400 °C, on the other hand, the resistivity increased in reversal. This tendency was apparent especially at thin region, i.e., around 10 nm (Figure S11, Supporting Information). To consider the effect of the process temperature before ALD and during ALD separately, we conducted additional experiments by changing the substrate loading temperature and heating up to the target process temperature (400 °C) in inert atmosphere. The results are shown in Figure S12 (Supporting Information), and the resistivity is relatively low as ≈13 µΩ cm when the loading temperature is up to 350 °C. However, the resistivity is high as 16.8 µΩ cm when loading the substrate at 400 °C even though the sheet resistance of underlayer TiN was subtracted properly. It means the partially oxidized TiN at 400 °C when loading into the ALD chamber also degraded the resistivity of deposited film itself (refer Section S2.1 in the Supporting Information for details). It also indicates the effect of air exposure is much more dominant than the effect of O_2_ exposure during ALD process.

**Figure 4:**
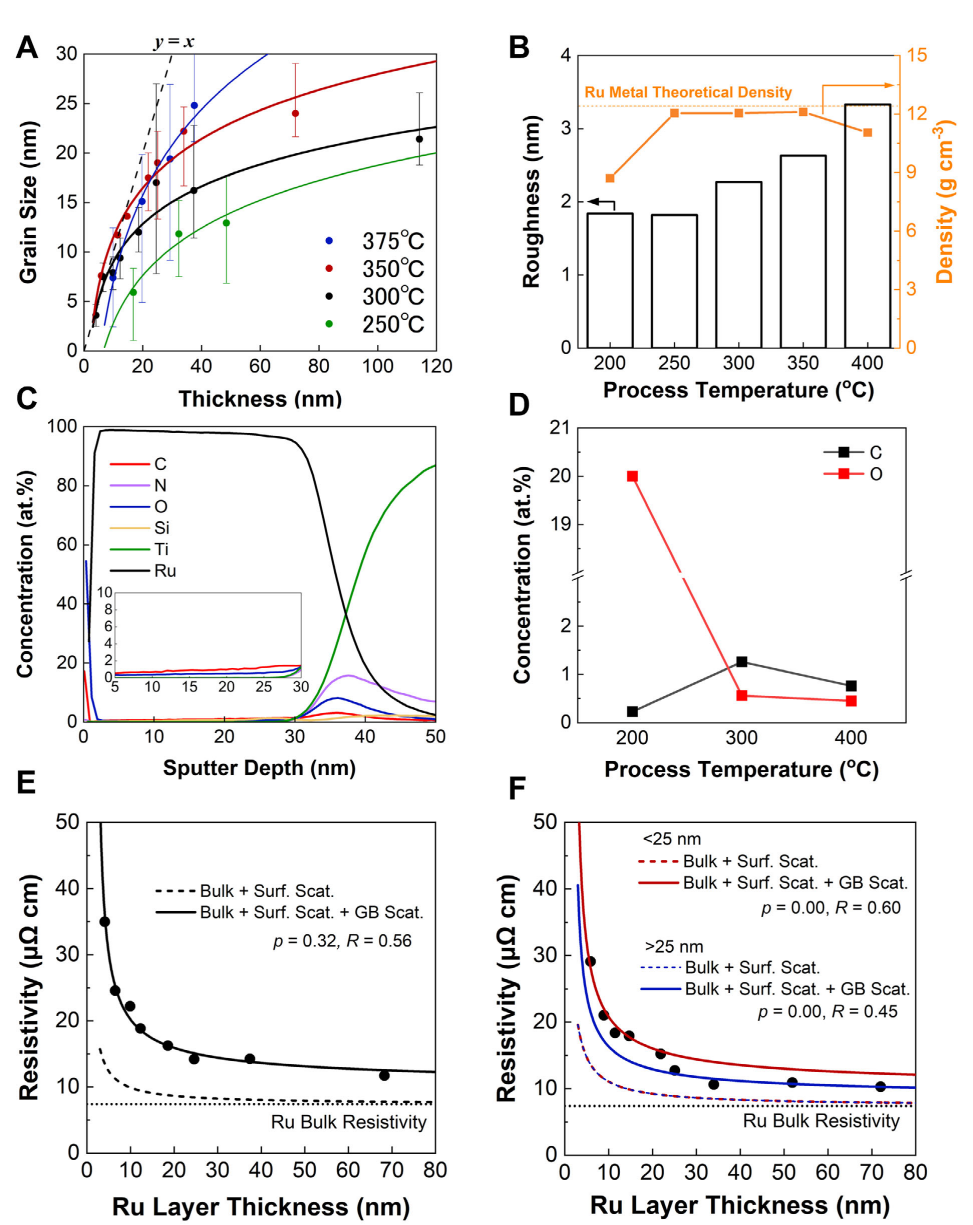
Characterization of ALD‐Ru thin films. A) Grain size evolution as a function of thickness at various process temperatures. B) Roughness and density estimated from X‐ray reflectometry (XRR). C) Secondary ion mass spectrometry (SIMS) depth profile of the ALD‐Ru film deposited at 300 °C. D) Representative value of carbon and oxygen impurities incorporated into the ALD‐Ru films evaluated by SIMS at various process temperatures. The contribution of bulk, surface scattering, and grain boundary scattering to the resistivity of ALD‐Ru thin films by adopting the FS–MS model fitted with *ρ*_0_ = 7.4 µΩ cm, *λ* = 6.6 nm E) deposited at 300 °C, F) at 350 °C.

Figure S13 (Supporting Information) shows the X‐ray diffraction patterns at different temperatures from 200 to 400 °C. All the samples were chosen with a thickness of around 30 nm. All peaks were identified as hexagonal‐closed‐packed (HCP)‐structured Ru metal, and no RuO_2_ peaks were observed despite using O_2_ as a reactant. Particularly in the low‐temperature range, such as 200 °C, the peaks were broad, and peak intensity was also low. Meanwhile, with the temperature increase, the peaks become sharper, indicating that crystallinity has improved. **Figure** **4**A shows the grain size derived from the Scherrer equation with deposited Ru thickness from 250 to 375 °C. Interestingly, we can draw the linear correlation as *y* = *x* between grain size *y* and deposited Ru thickness *x*, and the data at 350 °C are on the line at the <20 nm range. This means that the Ru nuclei growth is superior to nuclei creation and tends to form a spherical morphology to minimize the total surface energy at the initial stage of ALD at high temperatures according to Gibbs–Curie–Wulff's theorem.^[^ ^55^, ^56^ ^]^ On the other hand, grain size is small at relatively low temperatures, such as 250 °C, and is not on the *y* = *x* line, indicating nuclei creation is superior to nuclei growth. Ru atoms become easier to move and diffuse on the surface at higher temperatures, making it easy to reach the crystal growth points. On the other hand, lower temperatures make it hard to move and diffuse on the surface, creating a new nuclei that minimize the total surface energy locally. At 375 °C, the grain growth at the thin region is slightly recessed but almost the same as at 350 °C. It indicates that 350 °C is enough to overcome the barrier on the surface diffusion of Ru atoms. A slight recession might also be derived from decreased diffusion time due to the increased GPC. In thick regions such as >40 nm, the grain size depends on the process temperature, suggesting grain growth occurs during the ALD process. These larger grains probably contribute to lower resistivity as previously reports of Ru ALD.^[^ ^26^ ^]^

Figure 4B shows the roughness and density of deposited Ru films on TiN estimated from X‐ray reflectometry (XRR) with 200 cycles from 200 to 400 °C. The film roughness started increasing from 300 °C with a temperature increase. Higher temperature can improve the grain size; however, it degrades the surface roughness in the case of this O_2_ ALD process thus the process temperature needs to be chosen carefully. In terms of film density from XRR, the value was relatively low as 8.71 g cm^−3^ at 200 °C. From 250 to 350 °C, the density is almost the same as Ru metal (12.4 g cm^−3^), and the density slightly decreased to 11.05 g cm^−3^ at 400 °C. To conduct a further investigation on the film density with the deposition temperature, we performed secondary ion mass spectroscopy (SIMS) analysis at 200, 300, 400 °C (Figure 4C,D; Figure S14, Supporting Information). At 200 °C, the oxygen concentration in the film was quite high at 20%. On the other hand, the C content in the film was only 0.23%, which is close to the case of [Ru(TMM)(CO)_3_].^[^ ^28^ ^]^ It indicates that *p*‐cymene ligands were sufficiently removed from the oxidized Ru surface during precursor adsorption, as shown in Section 2.3 (also refer to Section S2.2 in the Supporting Information for details). At 300 °C, the deposited film is almost entirely composed of Ru metal, corresponding to the X‐ray diffraction (XRD) results. The amounts of impurities were 0.56% for O and 1.26% for C, showing high C content. The low O impurities seem to be due to the high process temperature, where many previously reported RuO_2_ ALD were conducted below 300 °C,^[^ ^37^, ^57^, ^58^, ^59^ ^]^ indicating the Ru─Ru metal bond is favorable compared to the Ru─O bond in terms of thermodynamic equilibrium at 300 °C. On the other hand, the C impurities are higher than [Ru(TMM)(CO)_3_] but still relatively lower than other hydrocarbon‐based Ru precursors, for example, <2% about [Ru(EtCp)_2_],^[^ ^22^ ^]^ 1.6% about DMPR.^[^ ^60^ ^]^ It is attributed to the ligands being composed solely of hydrocarbons, particularly benzene rings of *p*‐cymene, with a relatively high C/H ratio. At 400 °C, the amounts of impurities are 0.45% for O and 0.76% for C, slightly lower than at 300 °C. High process temperature makes it easier to decompose the high‐carbon‐content hydrocarbon ligands into lower‐carbon‐content sources such as CO and CO_2_ by virtue of the high reactivity with oxygen, preventing them from incorporating into the film. The lower C impurities also suggest that the thermal decomposition of the precursor did not occur at 400 °C. In terms of O impurities, the outermost surface and interface between the underlayer TiN had a high O concentration (Figure S14B, Supporting Information), supporting oxygen diffusion into the subsurface of Ru discussed in Section 2.2, and the partial oxidation of TiN before the ALD process discussed in this section.

Rapid thermal annealing was performed on deposited Ru film obtained at 300 °C with 300 cycles while H_2_ gas flowed for 10 min to obtain films with even lower resistivity. Figure S1A (Supporting Information) shows the resistivity values with different annealing temperatures from 400 to 700 °C. The resistivity slightly decreased up to 500 °C, and drastically reduced from 600 °C, reaching 8.65 µΩ cm at 700 °C, very close to the bulk resistivity of Ru (7.4 µΩ cm). This revealed that post H_2_ annealing with high temperature is effective for obtaining further low resistivity. Considering the thermal budget of interconnects,^[^ ^61^, ^62^ ^]^ it also emphasizes the advantage of high temperature ALD because the driving force of high temperature can be fully exploited during the deposition whose time is relatively longer than that of postanneal. Detailed discussions are assigned in Section S2.3 and Figures S15–S17 in the Supporting Information.

To further elucidate the contribution factor of this O_2_ ALD process to the resistivity of the as‐deposited films quantitatively, Fuchs and Sondheimer^[^ ^63^, ^64^ ^]^ and Mayadas and Shatzkes^[^ ^65^, ^66^ ^]^ models were adopted for this work. The equation is noted in Equation (2) as follows

(2) $$\rho =\rho_{0}+\rho_{0}\lambda \frac{3\left(1-p\right)}{4d}+\rho_{0}\lambda \frac{3R}{2D\left(1-R\right)}$$

(*ρ*_0_: bulk resistivity, *λ*: electron mean‐free path, *p*: surface scattering specularity coefficient, *d*: line width, *R*: grain boundary reflection coefficient, *D*: grain size)

Resistivity at 300 and 350 °C was considered to avoid the effect of apparent partial oxidation of TiN. Figure 4E,F shows the resistivity concerning the deposited Ru thickness with FS–MS models at 300 and 350 °C, respectively. When considering FS–MS models, the value of *ρ*_0_ was adopted as 7.4 µΩ cm and *λ* was adopted as 6.6 nm^[^ ^54^, ^67^ ^]^ as a constant, grain size *D* was adopted from the experimentally derived value from XRD using the Scherrer formula (Figure S18, Supporting Information). The least‐squares method was used to fit parameters, which are surface scattering specularity coefficient *p* and grain boundary reflection coefficient *R*. Experimental results were well fitted to the FS–MS model (Figure S19, Supporting Information). Fitted parameters *p* and *R* were 0.32 and 0.56 at 300 °C, respectively. At 350 °C, the value of *p* and *R* were 0.00 and 0.60, respectively, at thin regions (<25 nm), indicating surface scattering and grain boundary scattering were both slightly degraded. In thick regions such as >25 nm, even though the *p* is same as *p* = 0.00, the *R* drastically decreased as *R* = 0.45, resulting in low resistivity as 10.6 µΩ cm at 34 nm without postannealing. These coefficient values are almost within the range of previously reported values on Ru/SiO_2_,^[^ ^54^ ^]^ suggesting the deposited Ru layer shows typical Ru characteristics and that the fitting was successful. The *p*‐value decreases when the Ru surface is oxidized or covered by dielectrics,^[^ ^68^ ^]^ thus the *p* at 350 °C might reflect the chemical state of the oxidized Ru surface. This means that when adopting the O_2_ ALD process at a higher temperature, surface scattering becomes more prominent with low *p*. However, the contribution to the total resistivity is not so high (Figure S20, Supporting Information), for example, 10 µΩ cm at 10 nm when assuming the total resistivity is composed of bulk and surface scattering terms at 350 °C, as shown in Figure 4F.

Next, we will discuss the effect of grain boundaries on resistivity. To clarify the difference, hereinafter, we will distinguish between crystallite and grain. As shown in Figure 4F, the value of *R* decreased from 0.60 to 0.45 around 25 nm, suggesting crystallite boundary circumstances have changed at that point. A possible explanation is that a low‐resistance boundary was formed during the ALD process. Since the low‐energy grain boundary has lower resistance, while the random grain boundary has a high potential barrier, resulting in a higher electron reflection coefficient at random grain boundary.^[^ ^6^, ^16^ ^]^ One of the relatively low grain boundaries is the so‐called CSL, which is a 3D superlattice facing two crystals with shared atoms to a specific orientation. To evaluate CSL for these as‐deposited films and also annealed films, we conducted an EBSD analysis.

### 2.5 Advanced Crystallographic Analysis for Elucidating the Bottleneck to Achieve Lower Resistivity of ALD‐Ru

**Figure 5:**
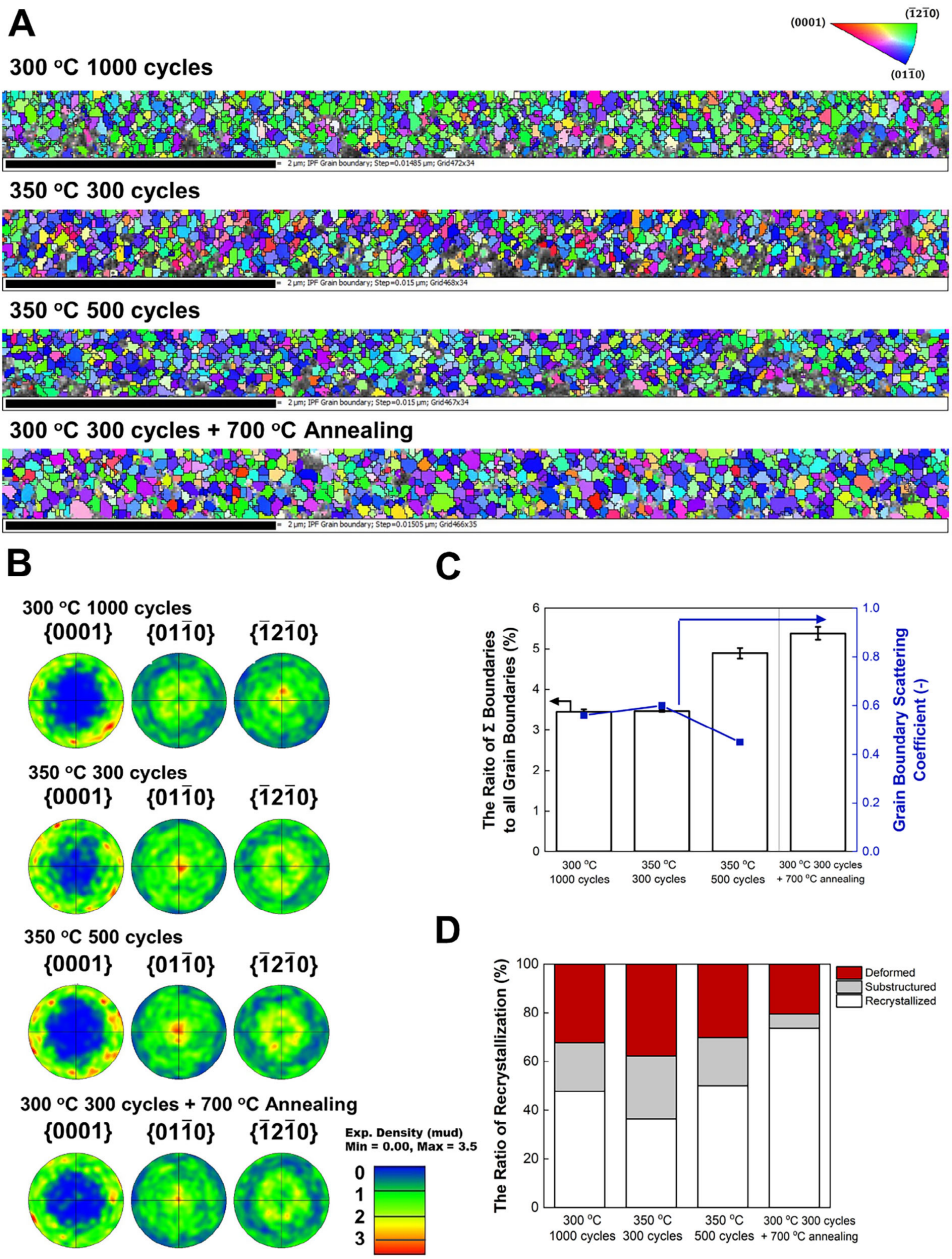
Electron backscatter diffraction (EBSD) of ALD‐Ru thin films for the samples of 300 °C 1000 cycles (114 nm Ru), 350 °C 300 cycles (22 nm Ru), 350 °C 500 cycles (34 nm Ru), and 300 °C 300 cycles after 700 °C annealing (34 nm Ru). A) Inverse pole figure maps. B) Pole figures. C) The ratio of coincidence site lattice (CSL) boundaries to the total grain boundaries and grain boundary scattering coefficient *R* derived from FS–MS model. D) The ratio of recrystallization.

**Figure** **5**A,B shows the inverse pole figure maps and pole figures of ALD‐Ru thin films derived from EBSD analysis, respectively (refer to Section S1.2 in the Supporting Information for details). The films have a specific preferred crystal orientation among all the analyzed samples as (01$\bar{1}$0) or ($\bar{1}$2$\bar{1}$0), which are parallel planes to the *c*‐axis of the Ru primitive lattice along the vertical direction to the substrate (Figure S21, Supporting Information). Also, (0001) oriented grains are tricky to observe from all the analyzed samples. Further discussion about this orientation preference is assigned in Section S2.4 and Figure S22 in the Supporting Information.

Since the lattice parameter of cubic TiN is 4.24 Å (PDF00‐038‐1420^[^ ^69^ ^]^), *c*‐axis of Ru [lattice parameter: 4.28 Å (PDF00‐006‐0663)^[^ ^69^ ^]^] is more preferable along the horizontal direction than the vertical direction to the substrate to resolve the lattice mismatch at the interface between TiN and Ru layers, thus the parallel plane to *c*‐axis is likely to be oriented along vertical direction. From these points, higher process temperatures make the grains grow larger while preserving their initial stable orientation, indicating crystallite growth occurs, thanks to sufficient thermal energies to overcome the diffusion barrier on the surface. This means higher temperatures have an advantage of getting larger and well‐crystallized grains with minimum efforts because it does not need to reconstruct or realign the crystals that have already been oriented. The inverse pole figure maps of 350 °C, 300 cycles, and 500 cycles have almost the same characteristics of (01$\bar{1}$0) preference; thus, they support the above interpretation. Moreover, this (01$\bar{1}$0) preference was also observed at postannealed sample despite the ($\bar{1}$2$\bar{1}$0) preference on the as‐deposited film (300 °C, 1000 cycles). It may reflect the lower surface energy of (01$\bar{1}$0) than that of ($\bar{1}$2$\bar{1}$0) in HCP Ru,^[^ ^70^ ^]^ probably being the same as (111) preference in face‐centered‐cubic (FCC)‐structured Au/SiO_2_ system with postanneal.^[^ ^71^ ^]^

Figure 5C shows the ratio of CSL to all the grain boundaries in the as‐deposited films at 300 and 350 °C, as well as postannealed thin films. This report regards Σ≦29 as CSLs derived from previously reported hexagonal crystal data.^[^ ^72^ ^]^ The detailed information is listed in Table S6 (Supporting Information). The ratio is remarkably higher by almost 40% increase at 350 °C 500 cycles than 300 °C 1000 cycles and 350 °C 300 cycles, which excellently corresponds to the trend of grain boundary scattering coefficient *R*. This result revealed that the CSL formation leads to reducing the grain boundary scattering with the mitigation of *R*, indicating more stable electron transmissions were achieved at grain boundaries. Since high temperatures promote the diffusion of Ru atoms, it is considered that the Ru atoms will get many more opportunities to form a Ru─Ru bonding with stable alignment, and will share the electrons between the grains to stabilize the surface, resulting in the formation of CSLs. The previous report also suggested the value of *R* on Ru/SiO_2_ thin films decreased after annealing,^[^ ^54^ ^]^ indicating a similar phenomenon occurred during the deposition on this work. In our experimental result of the annealed sample, the ratio of CSL is significantly higher than 300 °C, 1000 cycles. Since no clear EBSD signals could be obtained from the as‐deposited sample (300 °C, 300 cycles), it demonstrates that postannealing also encourages forming CSL boundaries. Recrystallization fractions derived from local misorientation (refer to Section S1.2 in the Supporting Information for details) were also improved after postannealing (Figure 5D). This can be interpreted that Ru atom alignment improved via dislocation inside the grains, and it can spread through the grain boundaries, which brings intergranular stress relaxation to enlarge grain (if misorientation angle is low) or to form CSL (if misorientation angle is middle or high) at high temperatures. Moreover, the impurities incorporated into the film may also affect the alignment because the impurities probably exist at the interstitial space of Ru crystals due to smaller atomic radii of C and O than Ru, causing lattice distortion and presumably, interfering the dislocation. It implies that high‐purity film is important for the better circumstances at grain boundaries as well as enlarged grains. The drastic impurities decrease with 700 °C annealing supports the above explanation, which may also be one of the reasons to achieve further low resistivity as discussed in Section S2.2 in the Supporting Information.

Even though the thermal acceleration of CSL formation as discussed above, on the contrary, the absolute percentage of CSL in Ru films was lower than that of other metals such as Cu and Al.^[^ ^73^, ^74^, ^75^ ^]^ It is probably due to the HCP crystal of Ru, having *a*‐axis and *c*‐axis with *c*/*a* = 1.583, which is slightly different from the ideal value as *c*/*a* = 1.633, resulting in difficulties in forming perfect and highly symmetric CSL like Σ3 in FCC crystals. Previous reports demonstrated that even if all the grains are composed of randomly oriented polycrystallites, CSLs account for 8.5% of total grain boundaries in the case of cubic crystals,^[^ ^76^ ^]^ which is higher than that of HCP metal with *c*/*a* = 1.581 (1.67%),^[^ ^77^ ^]^ implying difficulties in forming CSLs in HCP crystals. It is probably one of the reasons why the value of *R* in Ru is relatively high at ≈0.60 without the thermal acceleration, quite close to the typical value of random grain boundary *R* = 0.65–0.75.^[^ ^6^ ^]^ Although the CSL is hard to form in the Ru systems, however, the electric current chooses the easiest way to flow the electrons, like a parallel connection. Therefore, a small portion of CSL might affect all electrical characteristics once the circuit has been completed, resulting in a 20% decrease in grain boundary scattering contribution to total resistivity (Figure S20B, Supporting Information). The drastic change can be interpreted as the completion of a circuit that is composed of CSLs. Suppose the proper grain boundary engineering technique is explored and adopted for the Ru systems. In that case, there will be a possibility of reducing the value of *R* and resistivity further because the *R* strongly affects the Fermi surface of the metals.^[^ ^6^, ^16^ ^]^ Since the Fermi surface of Ru is anisotropic, unlike Cu,^[^ ^6^, ^67^ ^]^ only specific boundaries could achieve the less scattered electron transmission. The previous report also predicted the existence of particular CSLs with low *R* in the HCP Ru.^[^ ^78^ ^]^ Therefore, suitably designed grain boundary engineering might provide a solution for acquiring further low resistivity. Our experimental data show that the distribution of each CSL is almost the same between the four analyzed samples, regardless of the CSL ratio to all the grain boundaries (Figure S23, Supporting Information), although it generally depends on the deposition condition in the case of a thin layer.^[^ ^71^ ^]^ It may suggest that thermal acceleration primarily contributes to forming CSLs on this work. Further discussion is assigned in Section S2.5 in the Supporting Information. Meanwhile, the results show that more than 50% of total CSLs are composed of Σ7, 11, 13, indicating that low‐index CSLs are essential to form the low scattering boundaries. Low‐index CSLs are relatively highly symmetric; thus, it can be interpreted that electron transmission at the grain boundaries is likely not to disturb that much, resulting in the formation of low *R* boundaries also in Ru systems, as observed in both the experimental results and simulation results of other metals such as Cu.^[^ ^78^, ^79^, ^80^, ^81^, ^82^ ^]^

Comprehensive investigations are warranted to elucidate further the influence of individual CSL boundaries on electrical resistivity. While enlarging crystallite size presents an alternative strategy for reducing resistivity in Ru‐based thin film systems, the intrinsic 3D spherical growth behavior of HCP Ru, as discussed in Section 2.4, poses a significant challenge, particularly under low‐temperature processing conditions. Therefore, specialized grain engineering techniques tailored for Ru thin film architectures are essential. From this perspective, high‐temperature ALD processes offer a dual advantage: they facilitate the formation of larger crystallites and promote the emergence of energetically favorable CSL boundaries. This synergistic effect renders high‐temperature processing a compelling route for achieving low‐resistivity Ru thin films, aligning it as a robust candidate for scalable and durable ALD‐Ru interconnect technologies in advanced semiconductor applications.

## 3 Conclusions

In summary, we have established a novel small and simple molecular ligand structure Ru precursor that overcomes key limitations in ALD‐Ru process design for next‐generation interconnect applications. This precursor demonstrates exceptional thermal stability up to 400 °C, enabling high‐temperature ALD‐Ru processes that enhance film densification, grain growth, and electrical performance. With a high growth per cycle (≈1.28 Å cycle^−1^) and short incubation period (≈8 cycles) on TiN, the process achieves low resistivity values approaching bulk Ru down to 10.6 µΩ cm without annealing, and 8.65 µΩ cm after postdeposition hydrogen annealing. The newly synthesized Ru precursor also exhibits excellent substrate selectivity, with no Ru nucleation on SiO_2_ even after 1000 ALD cycles at 300 °C, supporting area‐selective integration strategies into the complex device architectures. These improvements are attributed to enhanced crystallinity, grain growth, and the formation of low‐energy coincidence site lattice grain boundaries, which reduce electron scattering revealed by microstructural analysis via EBSD, advanced aberration‐corrected UHR‐TEM/STEM, FS–MS modeling, and MLIP‐based energy profile calculations. Compared to previously reported Ru‐ALD processes, which often suffer from low growth rates, long incubation periods, and high film resistivity due to poor thermal stability, this new Ru precursor offers a balanced solution toward integrating low‐resistance, barrierless metal interconnects in advanced semiconductor nodes.

## 4 Experimental Section

In the attached as Section S1 (Supporting Information). The Experimental Section further contained the following subsections.

S1.1. Novel Ru Precursor‐Enabled ALD‐Ru Process Developments and Optimizations (Supporting Information)

S1.2. Advanced Characterization Tools for ALD‐Ru Thin Film Properties Evaluations (Supporting Information)

S1.3. Computational Analysis of ALD Surface Behavior and Thermal Stability of the Ru Precursor (Supporting Information).

Supporting Information

## Conflict of Interest

The authors declare no conflict of interest.

## Supporting information



Supporting Information

## Data Availability

The data that support the findings of this study are available from the corresponding author upon reasonable request.

## References

[1] G. E. Moore , in Proc. IEEE , IEEE, Piscataway, NJ, USA 1998.

[2] G. E. Moore , in 1975 Int. Electron Devices Meeting , IEEE, Piscataway, NJ, USA 1975, pp. 11–13.

[3] P. A. Packan , Science 1999, 285, 2079.

[4] E. Pop , S. Sinha , K. E. Goodson , Proc. IEEE 2006, 94, 1587.

[5] D. Josell , S. H. Brongersma , Z. Tőkei , Annu. Rev. Mater. Res. 2009, 39, 231.

[6] D. Gall , J. Appl. Phys. 2020, 127, 050901.

[7] C. Pan , A. Naeemi , IEEE Electron Device Lett. 2014, 35, 250.

[8] R. L. Graham , G. B. Alers , T. Mountsier , N. Shamma , S. Dhuey , S. Cabrini , R. H. Geiss , D. T. Read , S. Peddeti , Appl. Phys. Lett. 2010, 96, 042116.

[9] D. Gall , J. Appl. Phys. 2016, 119, 085101.

[10] P. S. Ho , K.‐D. Lee , S. Yoon , G. Wang , in *5th Int. Conf. Thermal Mechanical Simulation Experiments Microelectronics Microsystems*, 2004 *EuroSimE 2004. Proc*., IEEE, Brussels, Belgium 2004, pp. 15–16.

[11] Z. Tőkei , K. Croes , G. P. Beyer , Microelectron. Eng. 2010, 87, 348.

[12] J. Noguchi , IEEE Trans. Electron Devices 2005, 52, 1743.

[13] J. P. Gambino , T. C. Lee , F. Chen , T. D. Sullivan , in 2009 16th IEEE Int. Symp. Physical Failure Analysis Integrated Circuits , IEEE, Suzhou, Jiangsu, China 2009, pp. 677–684.

[14] D. C. Edelstein , in 2017 IEEE Int. Electron Devices Meeting (IEDM) , IEEE, San Francisco, CA, USA 2017, pp. 14.1.1–14.1.4.

[15] Z. Li , Y. Tian , C. Teng , H. Cao , Materials 2020, 13, 5049. 33182434 10.3390/ma13215049 PMC7664900

[16] J. H. Moon , E. Jeong , S. Kim , T. Kim , E. Oh , K. Lee , H. Han , Y. K. Kim , Adv. Sci. 2023, 10, 2207321. 10.1002/advs.202207321 PMC10427378 37318187

[17] K. Croes , C. Adelmann , C. J. Wilson , H. Zahedmanesh , O. V. Pedreira , C. Wu , A. Lesniewska , H. Oprins , S. Beyne , I. Ciofi , D. Kocaay , M. Stucchi , Z. Tokei , in 2018 IEEE Int. Electron Devices Meeting (IEDM) , IEEE, San Francisco, CA 2018, pp. 5.3.1–5.3.4.

[18] R. Bernasconi , L. Magagnin , J. Electrochem. Soc. 2019, 166, D3219.

[19] H. Wojcik , M. Junige , W. Bartha , M. Albert , V. Neumann , U. Merkel , A. Peeva , J. Gluch , S. Menzel , F. Munnik , R. Liske , D. Utess , I. Richter , C. Klein , H. J. Engelmann , P. Ho , C. Hossbach , C. Wenzel , J. Electrochem. Soc. 2011, 159, H166.

[20] V. Y. Vasilyev , N. B. Morozova , I. K. Igumenov , Russ. Chem. Rev. 2014, 83, 758.

[21] T. Aaltonen , M. Ritala , K. Arstila , J. Keinonen , M. Leskelä , Chem. Vap. Deposition 2004, 10, 215.

[22] O.‐K. Kwon , J.‐H. Kim , H.‐S. Park , S.‐W. Kang , J. Electrochem. Soc. 2004, 151, G109.

[23] S.‐S. Yim , D.‐J. Lee , K.‐S. Kim , S.‐H. Kim , T.‐S. Yoon , K.‐B. Kim , J. Appl. Phys. 2008, 103, 113509.

[24] S.‐H. Choi , T. Cheon , S.‐H. Kim , D.‐H. Kang , G.‐S. Park , S. Kim , J. Electrochem. Soc. 2011, 158, D351.

[25] T.‐K. Eom , W. Sari , K.‐J. Choi , W.‐C. Shin , J. H. Kim , D.‐J. Lee , K.‐B. Kim , H. Sohn , S.‐H. Kim , Electrochem. Solid‐State Lett. 2009, 12, D85.

[26] S. Yeo , S.‐H. Choi , J.‐Y. Park , S.‐H. Kim , T. Cheon , B.‐Y. Lim , S. Kim , Thin Solid Films 2013, 546, 2.

[27] S. Cwik , K. N. Woods , M. J. Saly , T. J. Knisley , C. H. Winter , J. Vac. Sci. Technol., A 2020, 38, 012402.

[28] Y. Kotsugi , S.‐M. Han , Y.‐H. Kim , T. Cheon , D. K. Nandi , R. Ramesh , N.‐K. Yu , K. Son , T. Tsugawa , S. Ohtake , R. Harada , Y.‐B. Park , B. Shong , S.‐H. Kim , Chem. Mater. 2021, 33, 5639.

[29] H. Li , D. B. Farmer , R. G. Gordon , Y. Lin , J. Vlassak , J. Electrochem. Soc. 2007, 154, D642.

[30] J. V. Hoene , R. G. Charles , W. M. Hickam , J. Phys. Chem. 1958, 62, 1098.

[31] K. Miyajima , T. Nagata , F. Mafuné , T. Tsugawa , R. Harada , Phys. Chem. Chem. Phys. 2025, 27, 9244. 40237158 10.1039/d5cp00200a

[32] W. Sari , T.‐K. Eom , S.‐H. Kim , H. Kim , J. Electrochem. Soc. 2011, 158, D42.

[33] S.‐J. Lee , S.‐H. Kim , M. Saito , K. Suzuki , S. Nabeya , J. Lee , S. Kim , S. Yeom , D.‐J. Lee , J. Vac. Sci. Technol., A 2016, 34, 031509.

[34] Y. Kotsugi , Y. Uchiyama , N. Yanatori , R. Harada , H. Nakagawa , in 2025 IEEE Int. Interconnect Technology Conf. (IITC) , IEEE, Busan, South Korea 2025, pp. 1–3.

[35] M. Knaut , M. Junige , M. Albert , J. W. Bartha , J. Vac. Sci. Technol., A 2012, 30, 01A151.

[36] A. Böttcher , H. Niehus , J. Chem. Phys. 1999, 110, 3186.

[37] H. J. Jung , J. H. Han , E. A. Jung , B. K. Park , J.‐H. Hwang , S. U. Son , C. G. Kim , T.‐M. Chung , K.‐S. An , Chem. Mater. 2014, 26, 7083.

[38] T. Aaltonen , A. Rahtu , M. Ritala , M. Leskelä , Electrochem. Solid‐State Lett. 2003, 6, C130.

[39] M. Popovici , B. Groven , K. Marcoen , Q. M. Phung , S. Dutta , J. Swerts , J. Meersschaut , J. A. Van Den Berg , A. Franquet , A. Moussa , K. Vanstreels , P. Lagrain , H. Bender , M. Jurczak , S. Van Elshocht , A. Delabie , C. Adelmann , Chem. Mater. 2017, 29, 4654.

[40] K. Knapas , M. Ritala , Crit. Rev. Solid State Mater. Sci. 2013, 38, 167.

[41] G. N. Parsons , R. D. Clark , Chem. Mater. 2020, 32, 4920.

[42] J.‐M. Lee , S.‐H. Lee , J. Oh , W.‐H. Kim , Mater. Lett. 2023, 333, 133574.

[43] J. K. Lodha , J. Meersschaut , M. Pasquali , H. Billington , S. D. Gendt , S. Armini , Nanomaterials 2024, 14, 1212. 39057888 10.3390/nano14141212 PMC11280396

[44] E.‐H. Cho , D. Kong , I. Cho , Y. Leem , Y. M. Lee , M. Kim , C. T. Nguyen , J. Y. Lee , B. Shong , H.‐B.‐R. Lee , Chem. Mater. 2024, 36, 8663.

[45] C. T. Nguyen , E.‐H. Cho , N. L. Trinh , B. Gu , M. Lee , S. Lee , J.‐Y. Lee , Y. Kang , H.‐B.‐R. Lee , Chem. Mater. 2023, 35, 5331.

[46] M. Junige , M. Löffler , M. Geidel , M. Albert , J. W. Bartha , E. Zschech , B. Rellinghaus , W. F. V. Dorp , Nanotechnology 2017, 28, 395301. 28837051 10.1088/1361-6528/aa8844

[47] S. H. Oh , J. M. Hwang , H. Park , D. Park , Y. E. Song , E. C. Ko , T. J. Park , T. Eom , T. Chung , Adv. Mater. Interfaces 2023, 10, 2202445.

[48] J. Lu , J. W. Elam , Chem. Mater. 2015, 27, 4950.

[49] N. Leick , S. Agarwal , A. J. M. Mackus , S. E. Potts , W. M. M. Kessels , J. Phys. Chem. C 2013, 117, 21320.

[50] N. Leick , S. Agarwal , A. J. M. Mackus , W. M. M. Kessels , Chem. Mater. 2012, 24, 3696.

[51] R. Gaur , L. Mishra , M. Aslam Siddiqi , B. Atakan , RSC Adv. 2014, 4, 33785.

[52] D. Zanders , J. Obenlüneschloß , J.‐L. Wree , J. Jagosz , P. Kaur , N. Boysen , D. Rogalla , A. Kostka , C. Bock , D. Öhl , M. Gock , W. Schuhmann , A. Devi , Adv. Mater. Interfaces 2022, 9, 2201709.

[53] Y.‐L. Chen , Y.‐Y. Fang , M.‐Y. Lu , P. Y. Keng , S.‐Y. Chang , Appl. Surf. Sci. 2023, 629, 157440.

[54] S. Dutta , K. Sankaran , K. Moors , G. Pourtois , S. Van Elshocht , J. Bömmels , W. Vandervorst , Z. Tőkei , C. Adelmann , J. Appl. Phys. 2017, 122, 025107.

[55] R. Li , X. Zhang , H. Dong , Q. Li , Z. Shuai , W. Hu , Adv. Mater. 2016, 28, 1697. 26678680 10.1002/adma.201504370

[56] G. Wulff , Z. Kristallogr. 1901, 34, 449.

[57] Y. Kim , M. Kim , Y. Kotsugi , T. Cheon , D. Mohapatra , Y. Jang , J. Bae , T. E. Hong , R. Ramesh , K. An , S. Kim , Adv. Funct. Mater. 2022, 32, 2206667.

[58] S. Yeo , J.‐Y. Park , S.‐J. Lee , D.‐J. Lee , J. H. Seo , S.‐H. Kim , Microelectron. Eng. 2015, 137, 16.

[59] D. Z. Austin , M. A. Jenkins , D. Allman , S. Hose , D. Price , C. L. Dezelah , J. F. Conley , Chem. Mater. 2017, 29, 1107.

[60] K. Kukli , J. Aarik , A. Aidla , I. Jõgi , T. Arroval , J. Lu , T. Sajavaara , M. Laitinen , A.‐A. Kiisler , M. Ritala , M. Leskelä , J. Peck , J. Natwora , J. Geary , R. Spohn , S. Meiere , D. M. Thompson , Thin Solid Films 2012, 520, 2756.

[61] C. S. Hwang , Atomic Layer Deposition for Semiconductors, Springer, New York, USA 2014, p 263.

[62] T. Nogami , O. Gluschenkov , Y. Sulehria , S. Nguyen , B. Peethala , H. Huang , H. Shobha , N. Lanzillo , R. Patlolla , D. Sil , A. Simon , D. Edelstein , N. Felix , J. Liu , T. Tabata , F. Mazzamuto , S. Halty , F. Roze , Y. Okuno , A. Uedono , in 2022 IEEE Symp. VLSI Technology Circuits (VLSI Technology Circuits) , IEEE, Honolulu, HI, USA 2022, pp. 423–424.

[63] E. H. Sondheimer , Adv. Phys. 2010, 1, 1.

[64] K. Fuchs , Math. Proc. Cambridge Philos. Soc. 1938, 34, 100.

[65] A. F. Mayadas , M. Shatzkes , Phys. Rev. B 1970, 1, 1382.

[66] A. F. Mayadas , M. Shatzkes , J. F. Janak , Appl. Phys. Lett. 1969, 14, 345.

[67] E. Milosevic , S. Kerdsongpanya , A. Zangiabadi , K. Barmak , K. R. Coffey , D. Gall , J. Appl. Phys. 2018, 124, 165105.

[68] S. S. Ezzat , P. D. Mani , A. Khaniya , W. Kaden , D. Gall , K. Barmak , K. R. Coffey , J. Vac. Sci. Technol., A 2019, 37, 031516.

[69] S. N. Kabekkodu , A. Dosen , T. N. Blanton , Powder Diffr. 2024, 39, 47.

[70] Y. Nanba , T. Ishimoto , M. Koyama , J. Phys. Chem. C 2017, 121, 27445.

[71] S. Kobayashi , H. Takagi , T. Watanabe , Philos. Mag. 2013, 93, 1425.

[72] R. Bonnet , E. Cousineau , D. H. Warrington , Acta. Crystllogr., Sect. A 1981, 37, 184.

[73] V. Randle , The Role of the Coincidence Site Lattice in Grain Boundary Engineering, CRC Press, Boca Raton, FL USA 1997, p. 128.

[74] M. P. Butrón‐Guillén , J. G. Cabañas‐Moreno , J. R. Weertman , Scr. Metall. Mater. 1990, 24, 991.

[75] R. V. Belluz , K. T. Aust , Metall. Trans. A 1975, 6, 219.

[76] D. H. Warrington , M. Boon , Acta Metall. 1975, 23, 599.

[77] D. H. Warrington , J. Phys. Colloq. 1975, 36, C4.

[78] S. Aboud , J. Huang , J. Cobb , T. Gunst , P. Asenov , T. Dam , R. Borges , in Design‐Process‐Technology Co‐optimization XV (Eds: C.‐M. Yuan , R.‐H. Kim ), SPIE, Bellingham, Washington 2021, p. 27.

[79] T.‐H. Kim , X.‐G. Zhang , D. M. Nicholson , B. M. Evans , N. S. Kulkarni , B. Radhakrishnan , E. A. Kenik , A.‐P. Li , Nano Lett. 2010, 10, 3096. 20608715 10.1021/nl101734h

[80] L. Lu , Y. Shen , X. Chen , L. Qian , K. Lu , Science 2004, 304, 422. 15031435 10.1126/science.1092905

[81] M. César , D. Liu , D. Gall , H. Guo , Phys. Rev. Appl. 2014, 2, 044007.

[82] N. A. Lanzillo , J. Appl. Phys. 2017, 121, 175104.

