# Supplementary material for: Small and Simple Molecular Structure Based Thermally Stable Ruthenium Precursor in Advancing Ruthenium ALD Process for Scaled Interconnect Metallization
Source: Adv Sci (Weinh). 2025 Nov 23;13(8):e19209. doi: 10.1002/advs.202519209 (PMC12884743; doi:10.1002/advs.202519209)
Supplement: Supplementary file 1 — Supporting Information [file ADVS-13-e19209-s001.docx]

Supporting Information

**Small and Simple Molecular Structure-based Thermally Stable Ruthenium Precursor in Advancing Ruthenium ALD Process for Scaled Interconnect Metallization**

Hideaki Nakatsubo, Debananda Mohapatra, Eun-Soo Lee, Jeongha Kim, Iaan Cho, Masato Iseki, Toshiyuki Shigetomi, Ryosuke Harada, Sang-Woong Na, Taehoon Cheon, Bonggeun Shong, and Soo-Hyun Kim*

**S1. Experimental Section**

*S1.1. Novel Ru Precursor-Enabled ALD-Ru Process Developments and Optimizations*

The Ru metal-organic precursor, [Ru(TMM)(*p*-cymene)] (TANAKA PRECIOUS METAL TECHNOLOGIES Co., Ltd., Japan), used in this work, was synthesized based on a previously reported procedure.^[1]^ Thermogravimetric analysis was conducted under N_2_ flow from ambient to 500 ^o^C at a rate of 5 ^o^C min^-1^ (STA 2500, NETZSCH). Thermal stability of [Ru(TMM)(*p*-cymene)] was evaluated using a traveling-wave-type ALD reactor (Lucida-D200, NCD Technology, Korea) before conducting a sequence of experiments. The precursor was vaporized in a bubbler-type canister heated to 80 ^o^C and was carried by N_2_ gas at a flow rate of 50 standard cubic centimeters per minute (sccm), supplying to the chamber from 300 to 400 ^o^C for 10min without any reactants. The amount of thermally decomposed Ru on SiO_2_/Si and TiN/SiO_2_/Si were evaluated using X-ray fluorescence (XRF, FT-160, FP methods, Hitachi, Japan).

[Ru(TMM)(*p*-cymene)]-O_2_ ALD was performed using the ALD reactor, and the process temperature was set to 300℃ unless otherwise specified. The precursor was also heated up to 80℃ and carried by N_2_ gas at a flow rate of 50 sccm. The flow rate of O_2_ gas was set to 200 sccm, and N_2_ gas was adopted for purging both precursor and reactant at a flow rate of 100 sccm. Unless otherwise specified, each pulse time was set to 10-10-10-10 s (precursor-purging-reactant-purging). The chamber pressure was adjusted to 1 Torr during precursor supply by setting the downstream valve opening. The base pressure in the vacuum state of the equipment was ~0.003 Torr, and the total pressure during the process was maintained at < 2.5 Torr. SiO_2_, TiN, and Ru were used as substrates; the sizes were 1 cm×1 cm. In the case of evaluating the step coverage, a Si-based trench patterned wafer was used with an aspect ratio of ~4 (Top width: ~115 nm, Bottom width: 65 nm, Trench depth: ~415 nm). A conformal c.a.10 nm TiN layer was deposited by ALD process using tetrakis(dimethylamino)titanium (TDMAT) and NH_3_ (pulse time: 1-10-1-10 s respectively) at 300 ^o^C, and a Ru film was sequentially deposited without any chamber venting, exposed to air. The aspect ratio after depositing TiN was also approximately 4.

*S1.2. Advanced Characterization Tools for ALD-Ru Thin Film Properties Evaluations*

The deposited substrates were analyzed ex-situ. Film thickness was quantified using an electron microscope (FE-SEM, SU8220, Hitachi, Japan). For SiO_2_ samples, XRF (FT-160, Hitachi High-Tech) was used for thickness quantification. The density and roughness of the films were confirmed by X-ray reflectometry (XRR, D8 DISCOVERY, Bruker, USA). Phase and crystallinity of the films were identified using grazing incidence angle X-ray diffraction (GIAXRD, D8 DISCOVERY, Bruker, USA) with Cu Kα radiation as an X-ray source. The incident angle was set to ω=3^o^. Film resistivity was measured using a four-point probe (CMT-100, AIT). The average value was adopted as a representative value by measuring the center of the substrate five times. Post-annealing was conducted using the rapid-thermal annealing (RTA) technique with H_2_ gas flow at 20 sccm for 10 min. The annealing temperature varied from 400 to 700 ^o^C. Compositional analysis was performed using secondary ion mass spectrometry (SIMS, Cameca IMS-7f using a 5 keV Cs^+^ beam, Korea Basic Science Institute). The cross-sectional view TEM (XTEM) equipped with a focused ion-beam technique for ALD-Ru thin film sample preparation evaluated by aberration-corrected ultra-high-resolution transmission electron microscopy (UHR-TEM, FEI Themis Z, Thermo-Fisher Scientific). Electron backscatter diffraction (EBSD) was employed to investigate the crystallographic orientations and to characterize grain boundary structures. All samples were prepared by ion milling (IM-4000, Hitachi) for 10 minutes to remove surface contaminants. Following sample preparation, the film surfaces were examined using a scanning electron microscope (SEM; MERLIN Gemini2, Zeiss, Germany) equipped with an EBSD detector (NordlysNano, Oxford Instruments, UK). EBSD measurements were conducted under SEM operating conditions with an accelerating voltage of 10 kV and a probe current of 1 nA. Diffraction patterns were acquired with a step size of 15 nm over a surface area of 7.0 × 0.5 µm. Commercial software packages—HKL CHANNEL 5 (Oxford Instruments, UK) and EDAX OIM (AMETEK, USA)—were used for the analysis of crystallographic orientation and grain boundary character distributions. Grain boundaries were determined with criteria of minimum misorientation angle as 5^o^. Coincidence site lattice (CSL) boundaries were identified based on the criteria proposed by R. Bonnet,^[2]^ and the Brandon criterion^[3]^ was applied to define the allowable deviation in misorientation angles for CSL boundary detection. Recrystallization fractions were determined through the internal procedure of HKL CHANNEL 5 Tago software. All the observed grains were categorized into 3 parts (recrystallized, substructured, deformed) with critical misorientation angle of grain as 5^o^ and that of sub-grain (*θ*_c_) as 1^o^. Deformed grains were defined as grains which have higher internal average misorientation angle within the grain than *θ*_c_. Substructured grains were chosen as grains which have lower internal average misorientation angle, but higher misorientation angle between sub-grains than *θ*_c_. All the remaining grains were classified as recrystallized grains.

*S1.3. Computational Analysis of ALD Surface Behavior and Thermal Stability of the Ru Precursor*

Machine learning interatomic potential (MLIP) based simulations were conducted to investigate the adsorption behavior of Ru(TMM)(p-cymene) on hcp Ru(0001),^[4]^ rutile RuO₂(110),^[5]^ and hydroxylated α-quartz SiO₂(001)^[6]^ surfaces. The PreFerred Potential (PFP),^[7,8]^ implemented in the Matlantis platform,^[9]^ was used to describe interatomic interactions as a pretrained universal neural-network potential covering 96 elements, trained on DFT data generated with VASP,^[10]^ the PBE exchange–correlation functional,^[11]^ a plane-wave cutoff of 520 eV, Gaussian smearing of 0.05 eV, and SCF tolerance of 10⁻⁴. D3(BJ) dispersion corrections were applied via the torch-dftd package.^[12-14]^ Geometry optimizations were performed using the L-BFGS algorithm in the Atomic Simulation Environment (ASE)^[15]^ with a force convergence threshold of 0.001 eV Å⁻¹. Additionally, DFT validation calculations for p-cymene desorption were performed using VASP with the PBE functional, under conditions consistent with the PFP setup, except for the use of a 3×3×1 Monkhorst–Pack k-point mesh. All slab models included a vacuum layer over 15 Å to eliminate interlayer interactions, with supercell dimensions of (16.25 × 16.25 × 23.54) Å³ for Ru, (12.68 × 12.45 × 31.98) Å³ for RuO₂, and (14.78 × 14.78 × 25.20) Å³ for SiO₂. The adsorption energy (*E*_ads_) was computed using equation S1 as follows;

*E*_ads_ = *E*_total_ – (*E*_slab_ + *E*_molecule_) (eq. S1)

where *E*_total_ is the energy of the adsorbate-slab system, *E*_slab_ is the energy of the clean slab, and *E*_molecule_ is the energy of the isolated precursor or ligand fragment. Ligand lifetimes (*τ* ) on each surface were estimated using an Arrhenius-type expression,

*τ* = *τ*_o_ × exp (*E*_des_ /*k*_B_*T*) (eq. S2)

where *E*_des_ is the desorption energy, *k*_B_ is the Boltzmann constant, *T* is the temperature set to 300 ℃, and *τ*_0_ is the attempt frequency. Lifetime comparisons were conducted using *τ* /*τ*_0_ to enable analysis independent of the specific choice of *τ*_0_.

DFT calculations were employed to evaluate the intrinsic thermal stability of Ru precursors by calculating the fragmentation energy using ORCA 5.0.3^[16]^ with the PBE0-D3BJ functional^[17–19]^ and def2-TZVP basis set.^[20]^ Fragmentation energy is defined here as the minimum energy required to dissociate a ligand from the Ru center, either through homolytic bond cleavage or through a hydrogen-rearranged pathway, depending on the ligand structure. Geometry optimizations were performed in the gas phase without symmetry constraints, and convergence was confirmed for both energy and gradient. The homolytic bond dissociation energy (BDE) was defined as

*BDE*_homolytic_ = *E*_L1_ + *E*_Ru(L2)_ -*E*_precursor_ (eq. S3)

where *E*_L1_ is the energy of the dissociated ligand, and *E*_Ru(L2)_ is the remaining Ru fragment. For ligands exhibiting rearrangement by hydrogen transfer, a rearranged BDE was also calculated as

*BDE*_rearranged_ = *E*_L1’_ + *E*_Ru(L2)(H)_*_n_* - *E*_precursor_ (eq. S4)

Here, L1' represents the rearranged ligand after hydrogen dissociation, and Ru(L2)(H)*n* denotes the Ru fragment with *n* hydrogen atoms redistributed.

**S2. Results and Discussion**

*S2.1. The effect of underlayer TiN oxidation for the deposited films and its resistivity at high temperatures*

As shown in Figure S10 (A), the effect of process temperature on the sheet resistance of underlayer TiN was also examined by only flowing 200 sccm O_2_ at 1Torr for 5 min, which is equivalent to 30 cycles in view of O_2_ supplying where a continuous Ru film might fully cover TiN surface as 4 nm. The sheet resistance of underlayer TiN gradually increased with the process temperature, indicating the surface of TiN was partially oxidized. However, there was almost no effect on the quantified value of deposited Ru film resistivity (Figure S10 (C)). Meanwhile, in our experimental results as shown in Section. 2.4, the effect of partial oxidation of the underlayer TiN before the ALD process is one of the domain reasons for high resistivity at higher process temperatures especially at 400 ^o^C. Interestingly, SIMS data (Figure S14 (B)) shows that an interface region at 400 ^o^C, which co-exists Ru and Ti, is relatively large compared to 200 ^o^C and 300 ^o^C. The possible reason is that the TiN surface became rough due to air exposure when loading the substrate. It corresponds to both XRR and SIMS results because a rough surface enlarges the interface region between TiN and Ru. An enlarged interface increases the apparent Ru layer thickness and decreases the net layer thickness of deposited Ru, resulting in the degradation of resistivity. As shown in Figure S12, the deposited Ru layer is slightly thicker when loading at 400 ^o^C; it can be interpreted as one of the effects of surface roughness increase. Figure S11 (A) shows the resistivity with process temperature around 10nm Ru. Compared with Figure S10(B), the process temperature, with the minimum resistivity, is lower in the case of 10nm Ru. It can be interpreted that the effect becomes stronger in the thin film because it mainly occurs at the interface between TiN and Ru layers, and the percentage of the effect on total resistivity becomes prominent in the thinner film. Moreover, grain size has no advantages at >350 ^o^C, as shown in Figure 4A; that is why the 350 ^o^C has minimum resistivity at 10 nm Ru. At thicker regions, those effects become relatively weak, and grain growth proceeds during the process, leading the process temperature having minimum resistivity to be shifted to a higher temperature; that is why the 375 ^o^C had the lowest resistivity around 30 nm Ru. Previous reports mentioned that surface roughness degrades resistivity^[21–23]^ and especially less than 10 nm^[22,24,25]^ with intense of the surface scattering. T. Zhou et al.^[23]^ have proposed the equation, which is a linear correlation between additional resistivity Δ*ρ* and surface roughness, and Δ*ρ* is inversely proportional to the layer thickness, indicating the surface roughness affects the resistivity, especially in thin regions. As reported by D. Gall,^[21]^ however, the contribution of surface roughness has certainly existed but not so high (<1%). In our experimental data show the degradation of surface scattering with low *p* at 350 ^o^C, however, it can be well interpreted as a change in the chemical state of oxidized Ru surface as discussed in Section 2.4. rather than the effect of surface roughness.

*S2.2. Oxidation of deposited Ru films at low temperatures*

In the case of Ru-O_2_ ALD, relatively low temperatures, such as around 200 ^o^C, high process pressure, and long O_2_ pulsing time encourage the formation of the RuO_2_ layer.^[26]^ Although no RuO_2_ diffraction peaks were shown in Figure S13, amorphous RuO_2_ was possibly formed partially. Since the density of RuO_2_ is 6.97 g cm^-3^, approximately 66% of the deposited Ru film is composed of RuO_2_, as estimated by the XRR result if all the oxygen is identified as RuO_2_. On the other hand, only 20% of the deposited film is composed of RuO_2_, which the SIMS estimate results with the same assumption. It indicates almost all the oxygens in the deposited film were not identified as RuO_2_ crystals but just incorporated into the structure of Ru metals like amorphous, corresponding to the results of XRD. The high GPC at 200 ^o^C cannot be entirely explained by the lattice expansion due to Ru oxide compound formation estimated from XRR and SIMS data, suggesting that precursor condensation occurred, as discussed in Section 2.2. The high resistivity at 200 ^o^C is discussed above was also attributed to this O impurities.

*S2.3. The annealing effect to deposited ALD-Ru thin films*

Figure S15 (B) shows the XRD results at each annealing temperature, and further peak sharpening was observed above 600 ^o^C, corresponding to the resistivity trend. Interestingly, Ru (002) diffraction peak disappeared above 600 ^o^C annealing, indicating reconstruction of crystal orientation. We additionally conducted Plane-View SEM to quantify the grain size by converting each area to an equivalent circle (Figure S16). The grain size distribution became slightly broader at 500 ^o^C compared to the as-deposited one, indicating partial grain growth occurred. Still, the overall grain size remains almost the same (center of distribution: 28.6 nm). At 700 ^o^C, the center of the grain size distribution significantly increases to 57 nm. The grain boundary also turned clear from the SEM image. It indicates that after H_2_ annealing at high temperatures, it becomes easier to move Ru atoms and reconstruct the grain with grain growth. Figure S17 (A)-(C) show the SIMS data after annealing. The amounts of impurities remained almost unchanged at 500 ^o^C but decreased with annealing at 700 ^o^C. Post-H_2_ annealing also promotes the removal of impurities from the deposited film, which might contribute to reducing resistivity to some extent.

*S2.4. Orientation preference of deposited Ru thin films on TiN*

GIDXRD results, as seen in Figure S13 and Figure S15 (B), show similar characteristics, the disappearance of Ru (0002) diffraction peak at 350 ^o^C samples and after annealing at 700 ^o^C. Even though the direction of a normal vector in GIDXRD differs from that of EBSD, the differences are estimated to be approximately just 20 degrees in the case of Ru (0002) peak measurement. The pole figures in Figure 5B shows the absence of Ru (0002) oriented grains even if tilted by 30 degrees, thus the results of EBSD and GIDXRD correspond in the case of Ru (0002) peaks in the range of 0-20 degrees titled, indicating extreme difficulties of c-axis Ru growth along the vertical direction to the substrate. Interestingly, the intensity of Ru (0002) GIDXRD peaks for 300 ^o^C deposition films is almost constant during the process (Figure S22). As discussed in Section 2.4, higher temperatures make the Ru diffusion easier; thus, many more Ru atoms can move to the energetically stable growth points. The small yet constant Ru (0002) peak at 300 ^o^C could be regarded as the Ru diffusibility difference on TiN and Ru at the initial nucleation stage.

*S2.5. Grain growth and the distribution of each coincidence site lattice (CSL)*

The driving force of crystal growth is generally interpreted as the reduction of the total energy in the system.^[27,28]^ It is derived from several factors such as surface energy^[29]^ or internal stress.^[30]^ The prime internal stress at the initial stage of deposition is the lattice mismatch between substrate and deposited atoms.^[28]^ In our experimental results show that this mismatch can be suppressed by adopting high process temperatures. After few layer deposition, the main factor of internal stress transitions from lattice mismatch to the location of deposited atoms.^[28]^ As a principle of Wulff's theorem,^[31]^ the crystal growth is mainly governed by the surface energy of the crystal plane from the thermodynamic perspective, thus we may control the crystal growth behavior with carefully tuning the deposition condition. With stacked internal stress during the process will be released through the dislocation^[27,30]^ when sufficient energy is supplied such as post-anneal treatment because the grain boundary characteristic in the system is metastable.^[29]^ Since this work adopted high process temperature, the location of atoms have already been aligned effectively except the impurities interference^[32]^ supported by the clear EBSD results (Figure 5A). In general, spread of the dislocation through the grain boundaries needs much more energy compared to the dislocation inside the grain.^[30]^ Thus, only higher temperatures such as > 600 ^o^C exhibit significant contribution to the crystallographic evolution because post-anneal treatment time is limited. As discussed in Section 3.5, grain enlargement proceeds if the misorientation angle is low, and CSL formation proceeds if the misorientation angle is middle or high.^[33]^ Therefore, post-annealing at 700 ^o^C supplied enough energy to spread the dislocation through the grain boundaries, thus it exhibits both grain growth and CSL formation as shown in Figure S16 and Figure 5C, respectively. Consequently, the formation of CSL is affected by many factors, not only the effect of substrate,^[29]^ crystal phase of deposited film^[27]^ and its orientation,^[33]^ but also deposition conditions,^[29]^ the way to stack internal stress^[30]^ and so on.


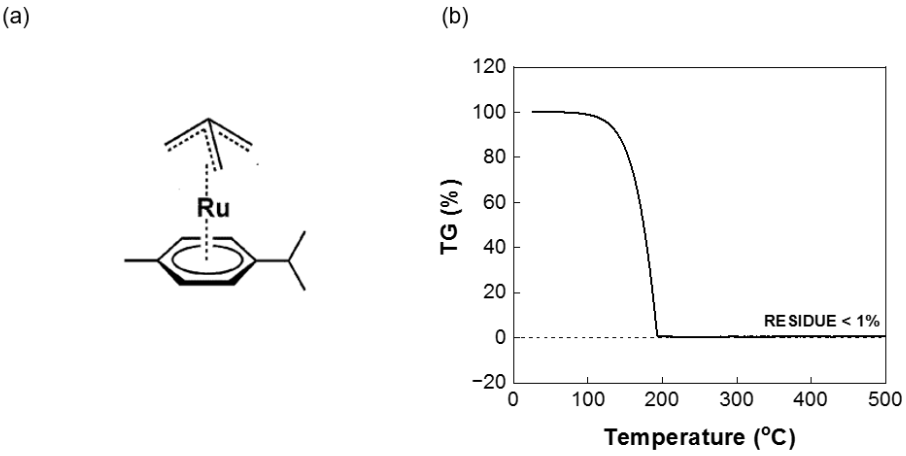


**Figure S1**. (A) Molecular structure of [Ru(TMM)(*p*-cymene)] Ru precursor (B) TG Curve for the Ru precursor.

**
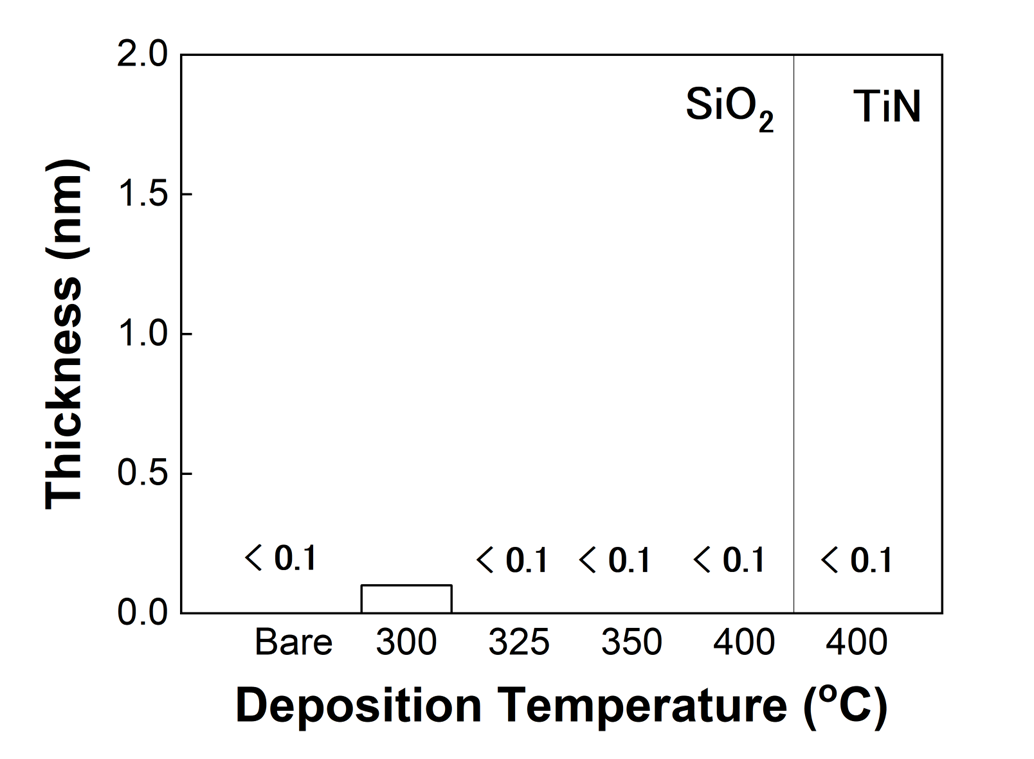
**

**Figure S2.** The amount of Ruthenium deposited on SiO_2_ and TiN substrates evaluated by X-ray fluorescence (XRF), supplying [Ru(TMM)(*p*-cymene)] for 10 minutes without any reactants at various temperatures.

**
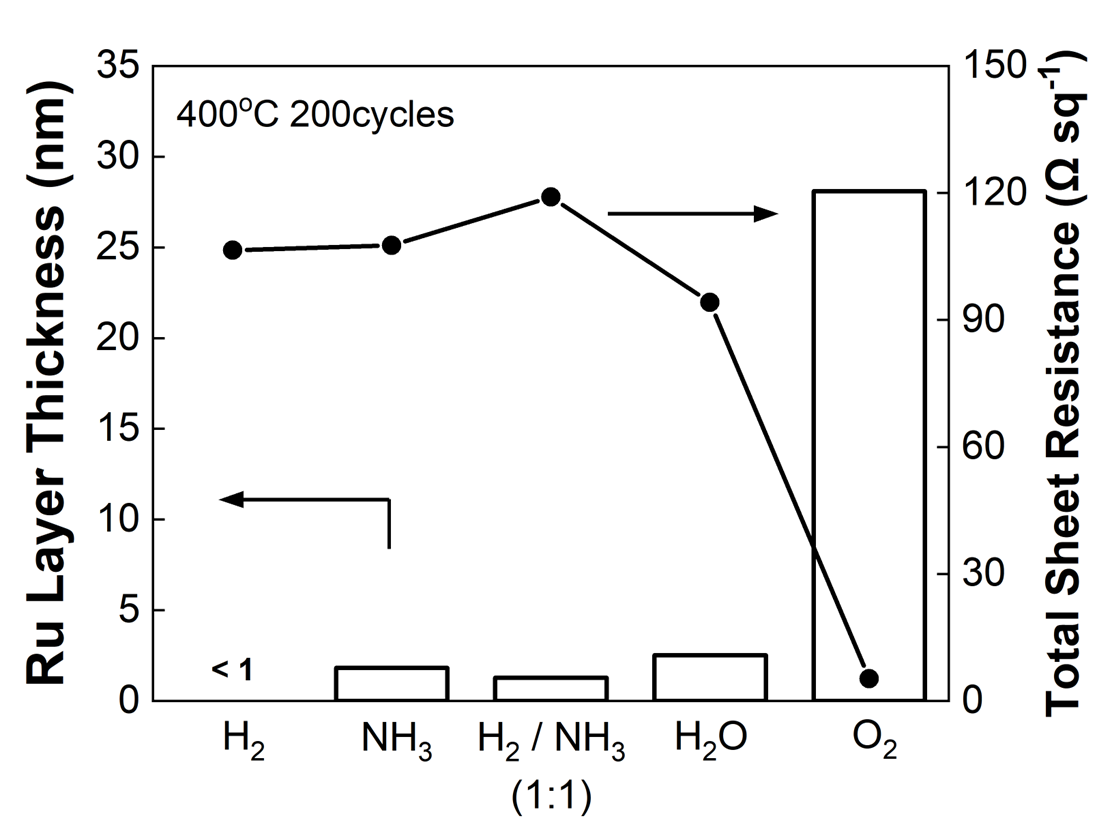
**

**Figure S3.** Deposited Ruthenium film thickness and total sheet resistance on TiN substrate for the selection of a reactant at 400 ^o^C deposition.


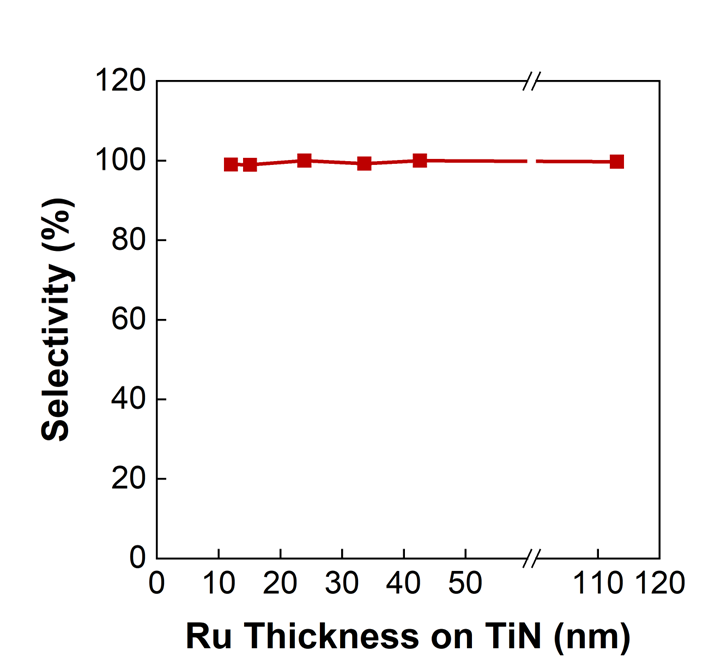


**Figure S4**. Selectivity on TiN v.s. SiO_2_ surface with deposited Ru thickness on TiN for the Ru precursor.


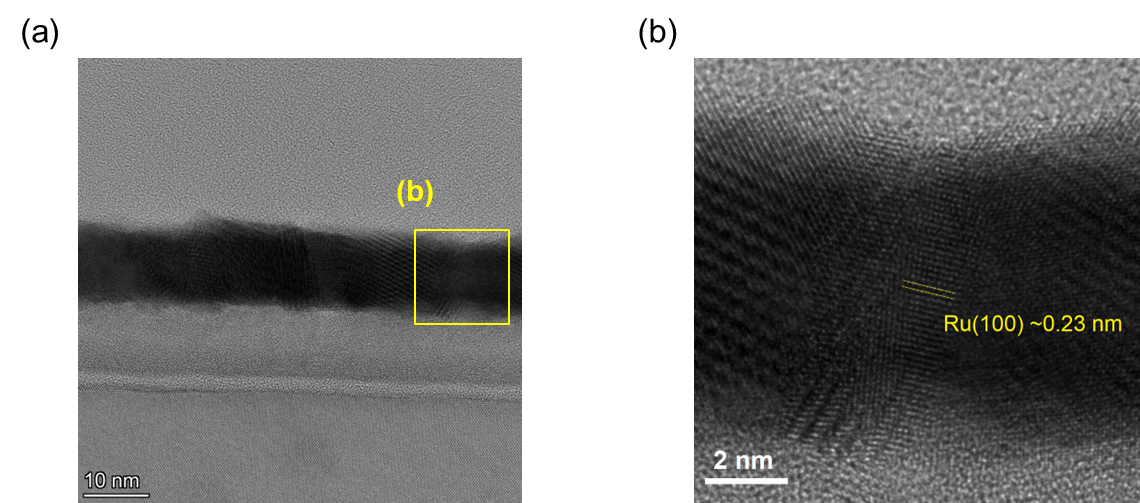


**Figure S5.** High-resolution images of cross-sectional view transmission electron microscopy (XTEM) with around 10nm Ru film on TiN deposited trench. (A) Top of the trench. (B) Magnified view.


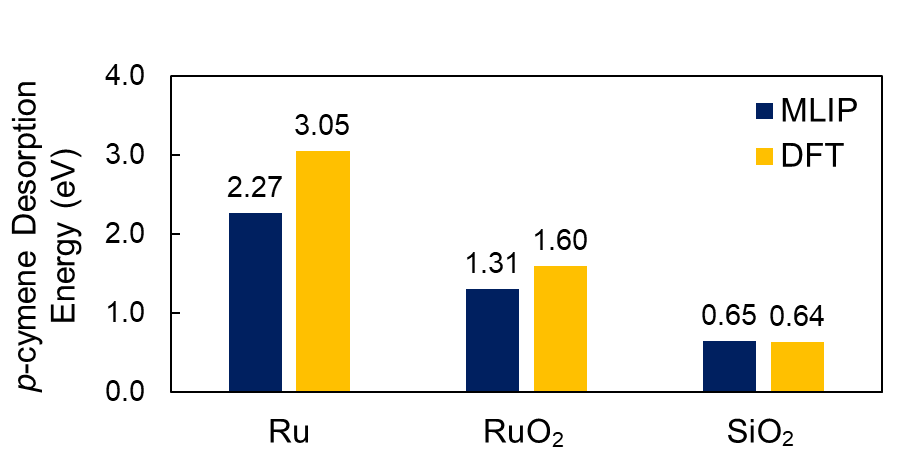


**Figure S6.** Comparison between MLIP and DFT calculated *p*-cymene desorption energies on Ru(0001), RuO_2_ (110), and hydroxylated SiO_2_ (001).


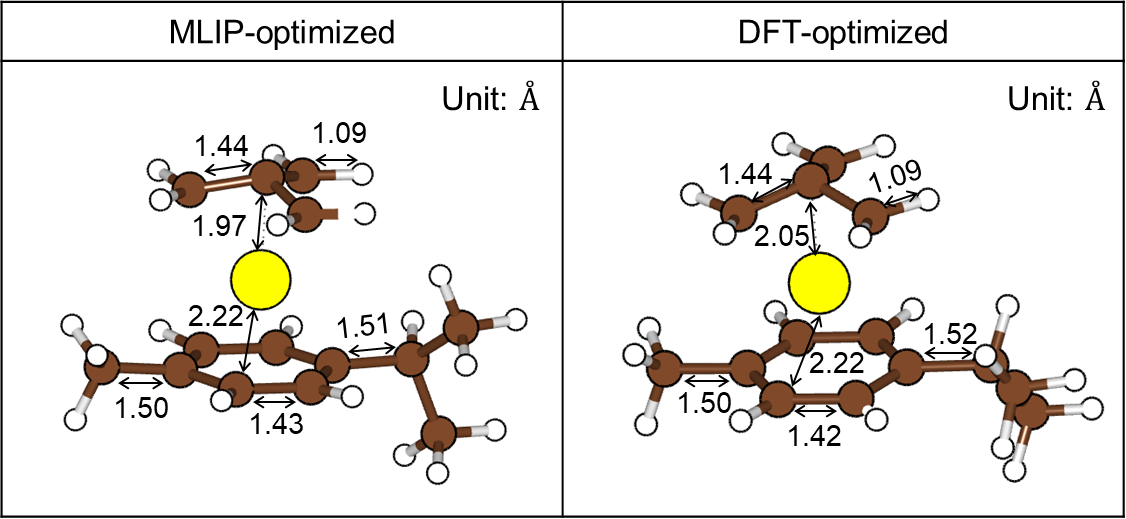


**Figure S7.** Comparison of internal bond lengths of the [Ru(TMM)(*p*-cymene)] precursor optimized with MLIP and DFT used in this study


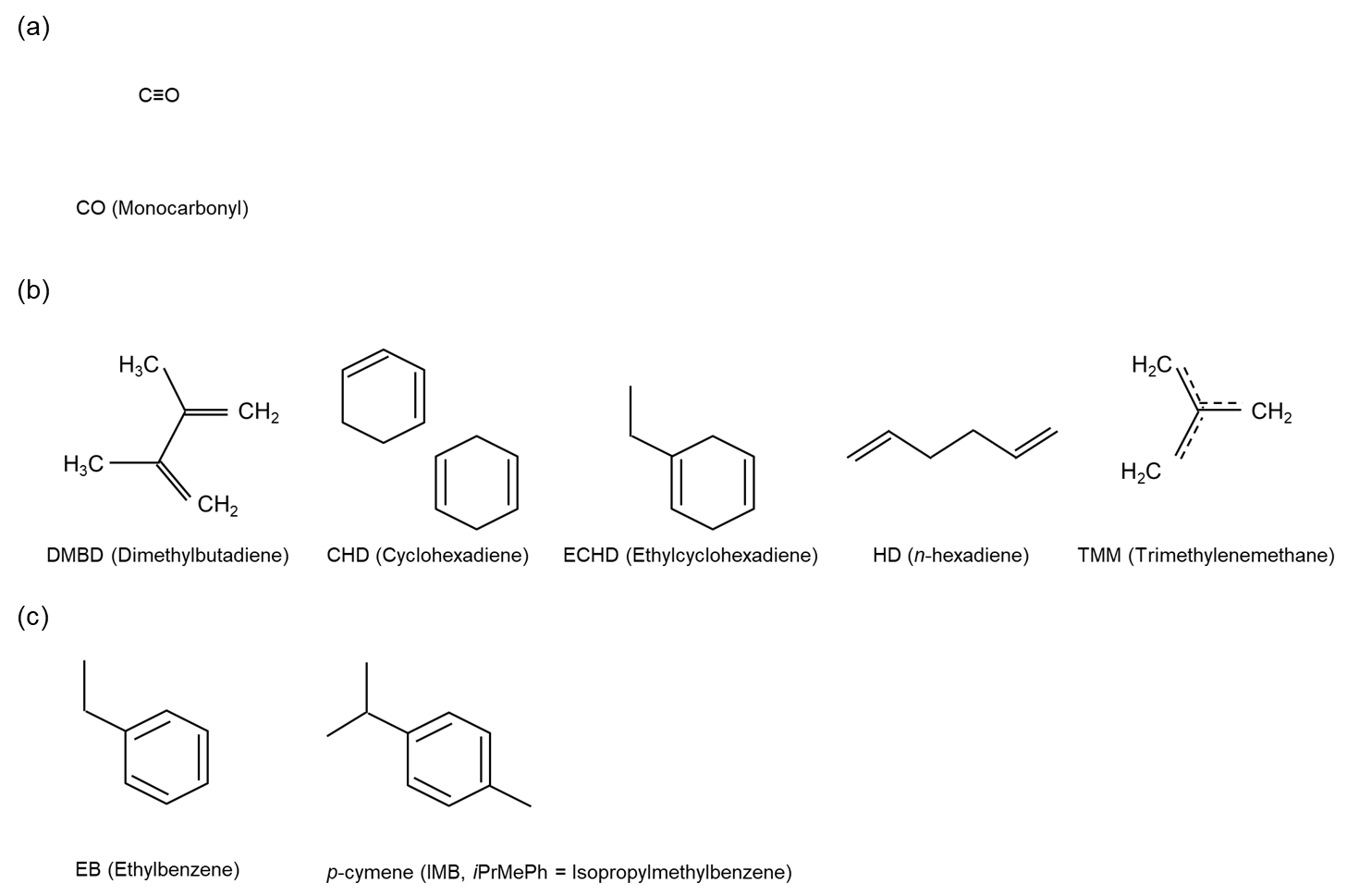


**Figure S8**. The chemical structure of different ligands and their abbreviations composing experimentally reported Ru(0) or Ru(+/0) precursors; (A) 2 electrons donated ligands, (B) 4 electrons donated ligands. (C) 6 electrons donated ligands.


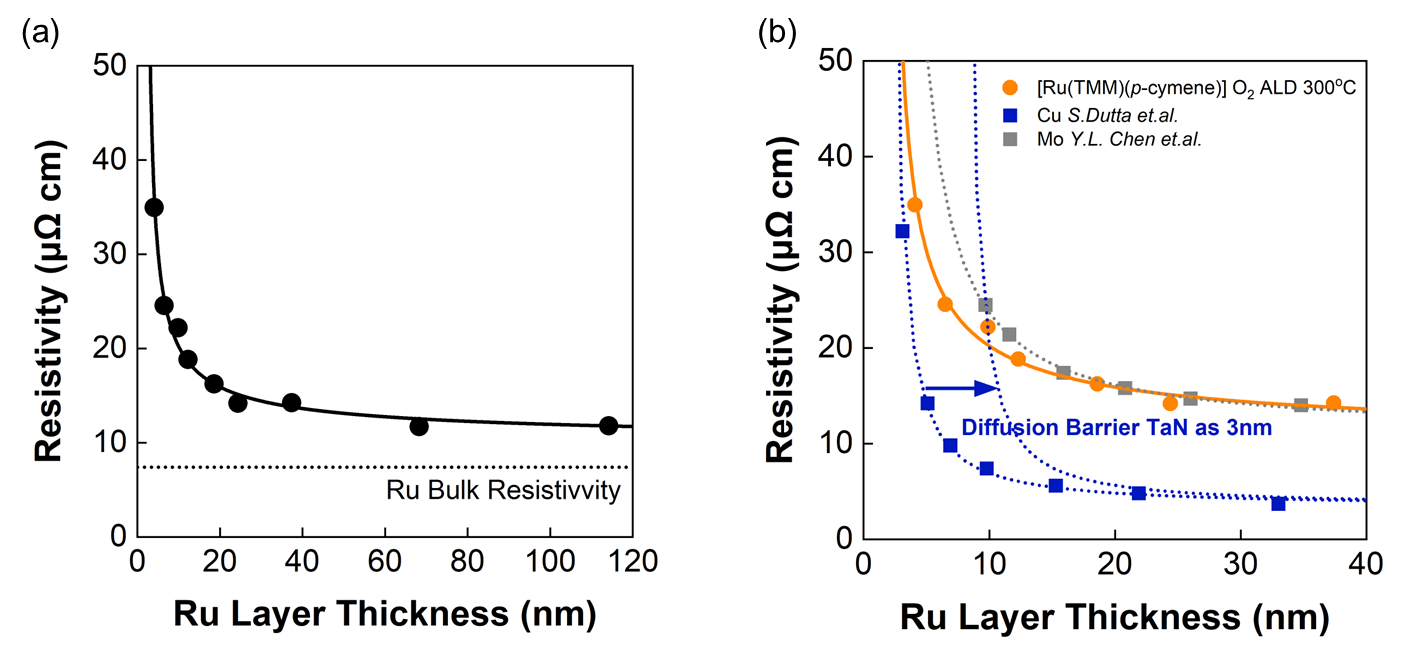


**Figure S9**. Resistivity of deposited ALD-Ru thin films at 300 ^o^C deposition (A) As a function of ALD-Ru film thickness (B) Resistivity comparison to Mo and Cu/TaN films especially at thin regions.


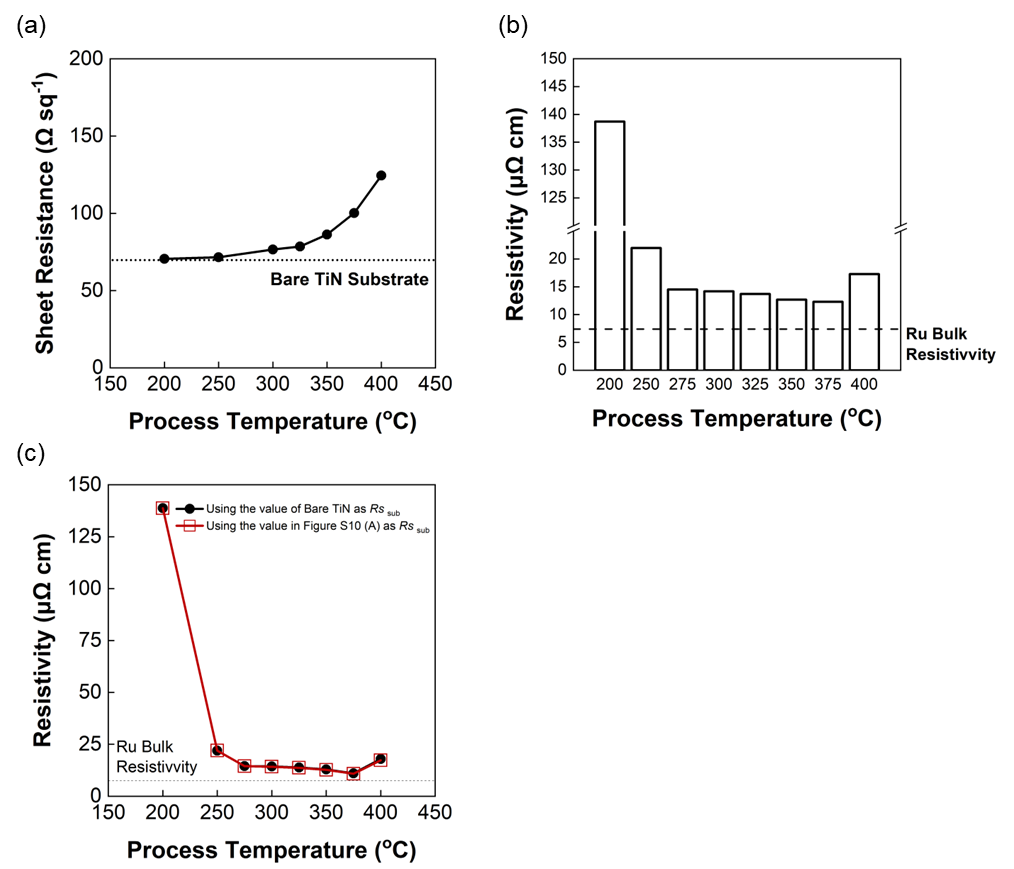


**Figure S10**. (A) The effects of high process temperature to sheet resistance of TiN substrate before the ALD process; 200 sccm O_2_ flow treatment under 1 Torr for 5 minutes at various temperatures. (B) Resistivity of deposited ALD-Ru thin films (around 30nm Ru) as a function of the process temperatures where the values in Figure S10 (A) were adopted as the sheet resistance of underlayer. (C) The effect of adopted underlayer sheet resistance to the resistivity of around 30 nm Ru films.


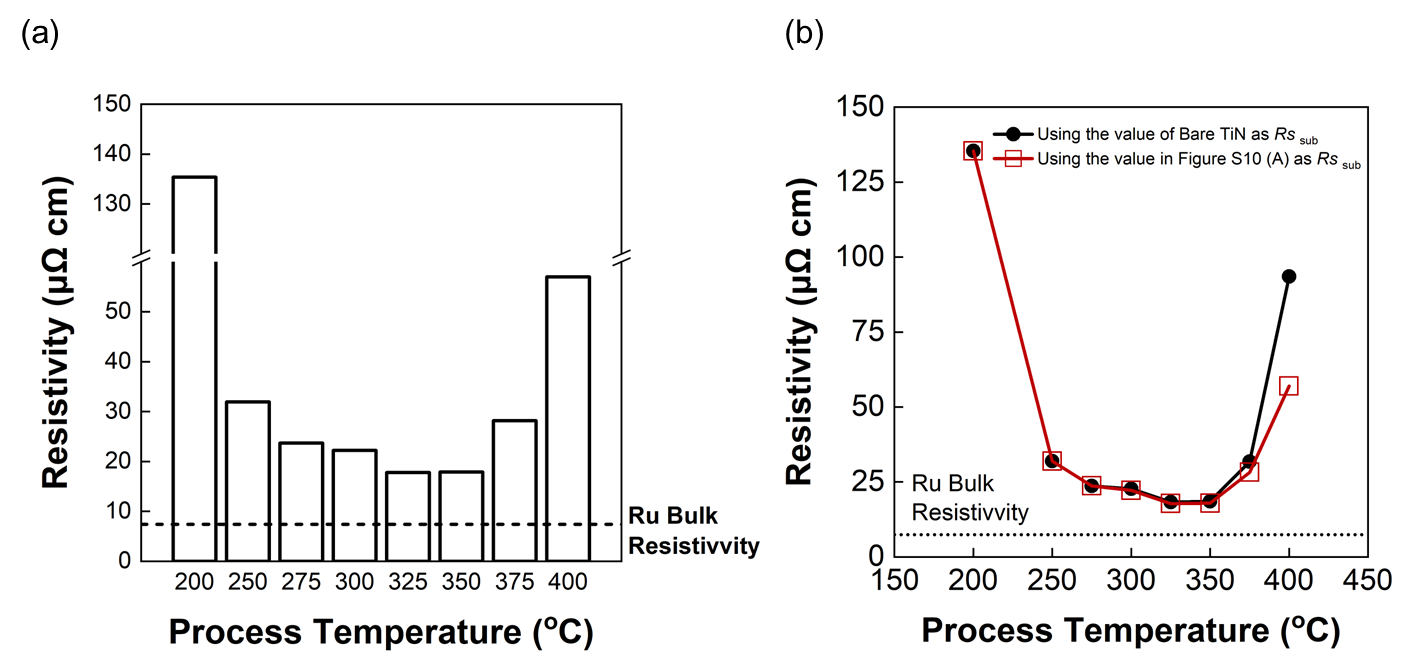


**Figure S11**. Resistivity of deposited ALD-Ru thin films as a function of the process temperatures (A) Around 10 nm Ru films where the values in Figure S10 (A) were adopted as the sheet resistance of underlayer (B) The effect of adopted underlayer sheet resistance to the resistivity of around 10 nm Ru films.


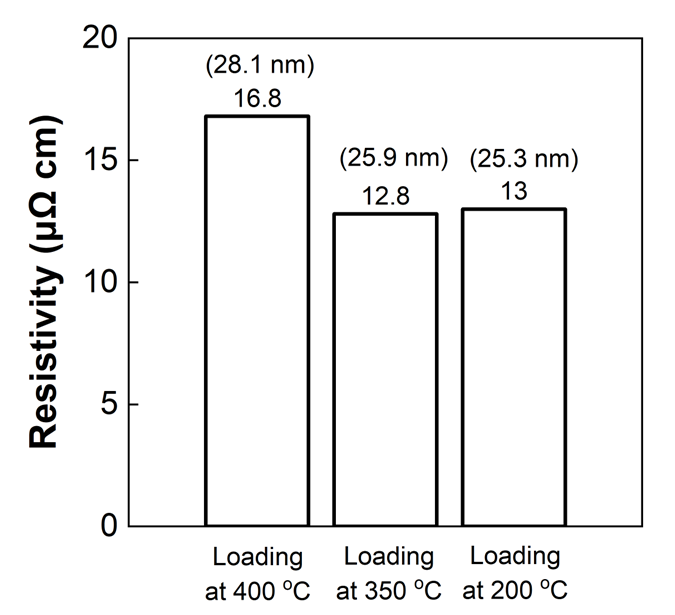


**Figure S12.** Resistivity of ALD-Ru films at 400 ^o^C deposition with 200 cycles and various substrate loading temperatures where the substrates were under the inert atmosphere during temperature elevation to 400 ^o^C. The sheet resistance of TiN was adopted the value in Figure S8 (A) to each loading temperature (the value in the parentheses represents deposited Ru thickness evaluated by scanning electron microscopy).


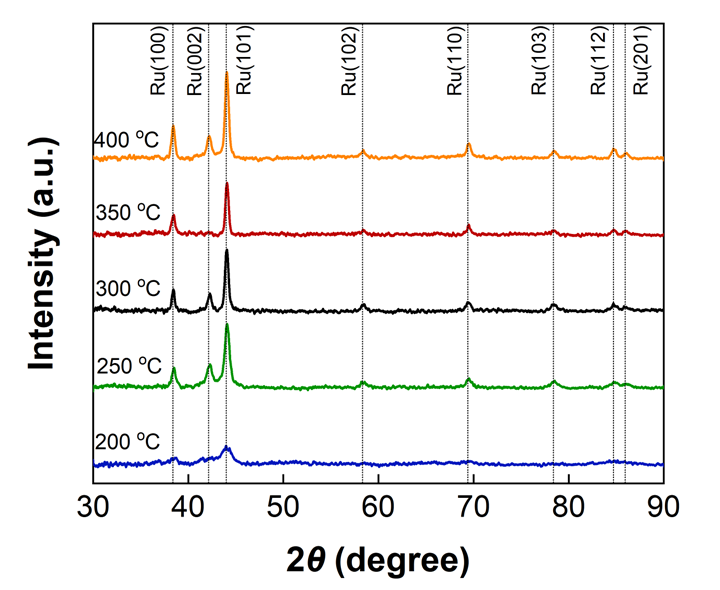


**Figure S13**. Diffraction peaks of grazing incidence angle X-ray diffraction (GIDXRD) on the ALD-Ru thin films with various process temperatures.


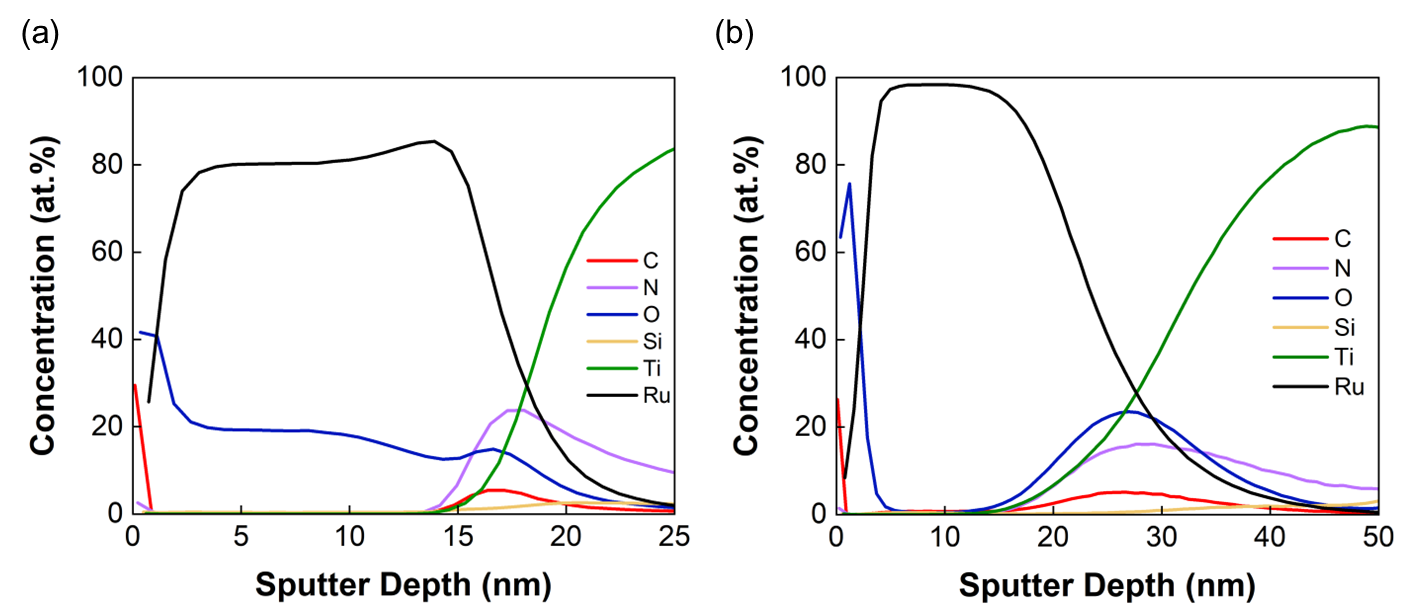


**Figure S14.** Depth profile for the ALD-Ru thin films by secondary ion mass spectrometry (SIMS) (A) At 200 ^o^C deposition (B) At 400 ^o^C deposition.


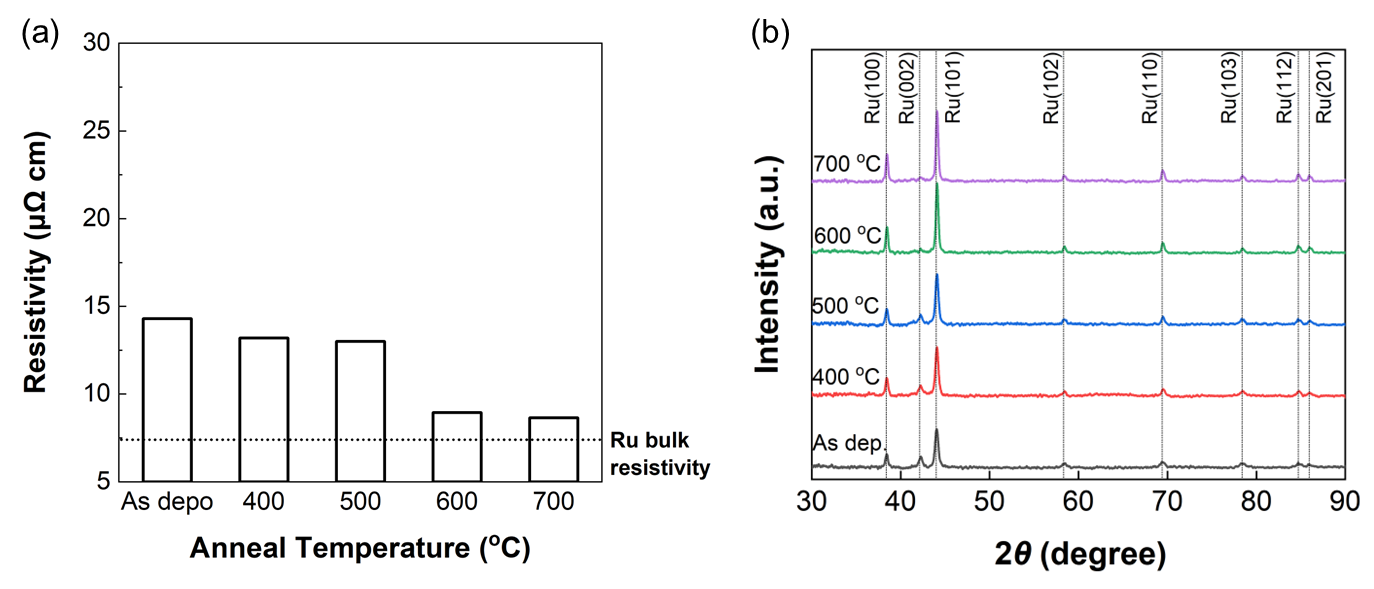


**Figure S15.** Properties of ALD-Ru thin films at 300 ^o^C deposition with 300 cycles after rapid thermal annealing (RTA) under H_2_ flow for 10 minutes (A) Resistivity (B) grazing incidence angle X-ray diffraction (GIDXRD) peaks.


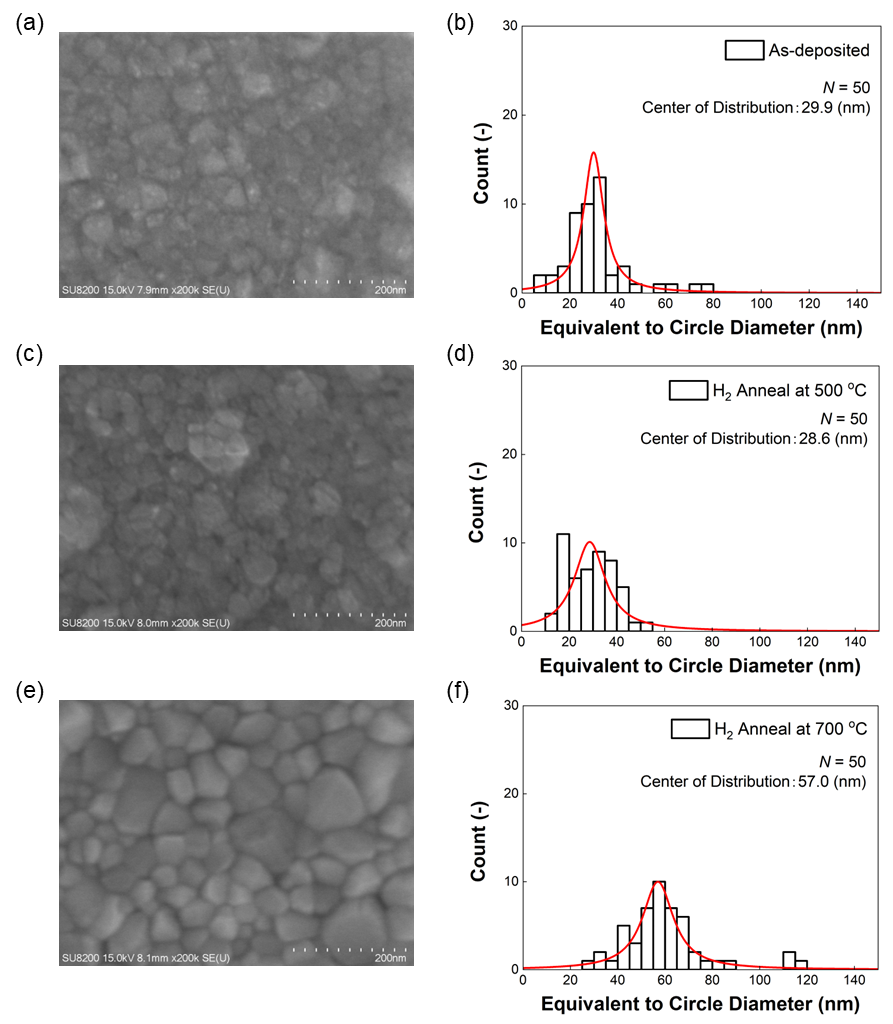


**Figure S16.** Top view SEM images of ALD-Ru thin films and grain size distributions (A), (B) As deposition (300 ^o^C 300 cycles) (C), (D) After 500 ^o^C anneal (E), (F) After 700 ^o^C anneal.


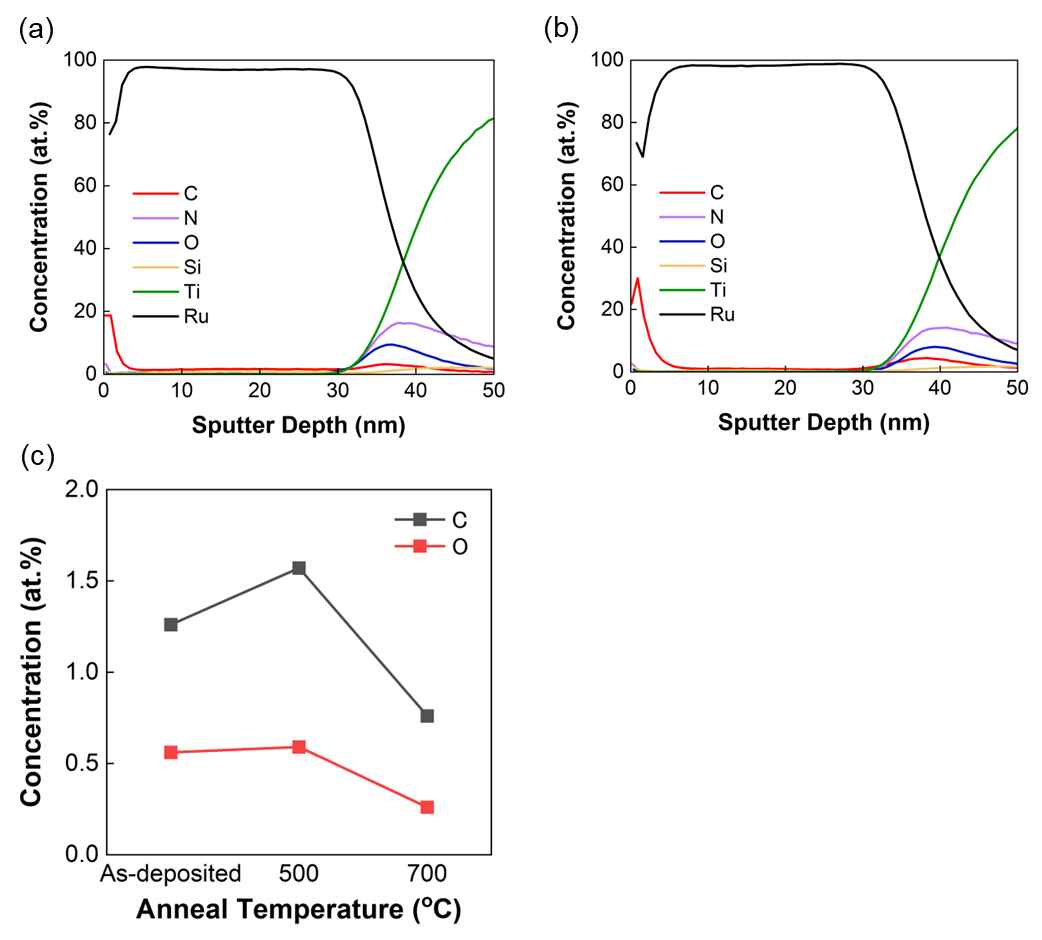


**Figure S17**. Composition analysis for the ALD-Ru thin films by secondary ion mass spectrometry (SIMS) after H_2_ anneal (A) Depth profile after 500 ^o^C anneal (B) Depth profile after 700 ^o^C anneal (C) Representative value of Carbon and oxygen impurities incorporated into the annealed ALD-Ru films.


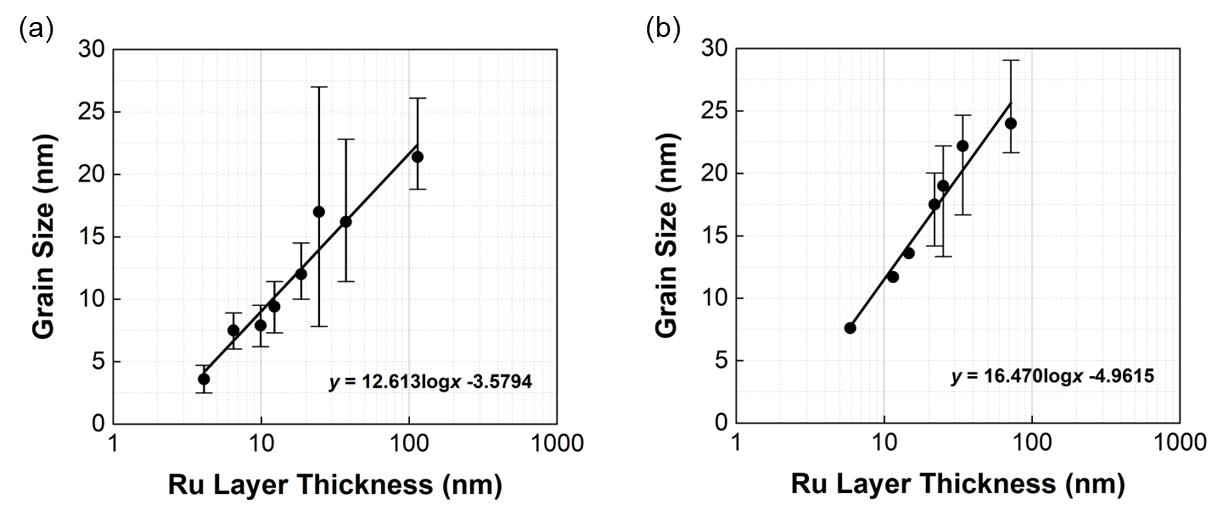


**Figure S18**. Logarithmic relation between grain size evaluated from GIDXRD and thickness of ALD-Ru thin film (A) At 300 ^o^C deposition (B) At 350 ^o^C deposition.


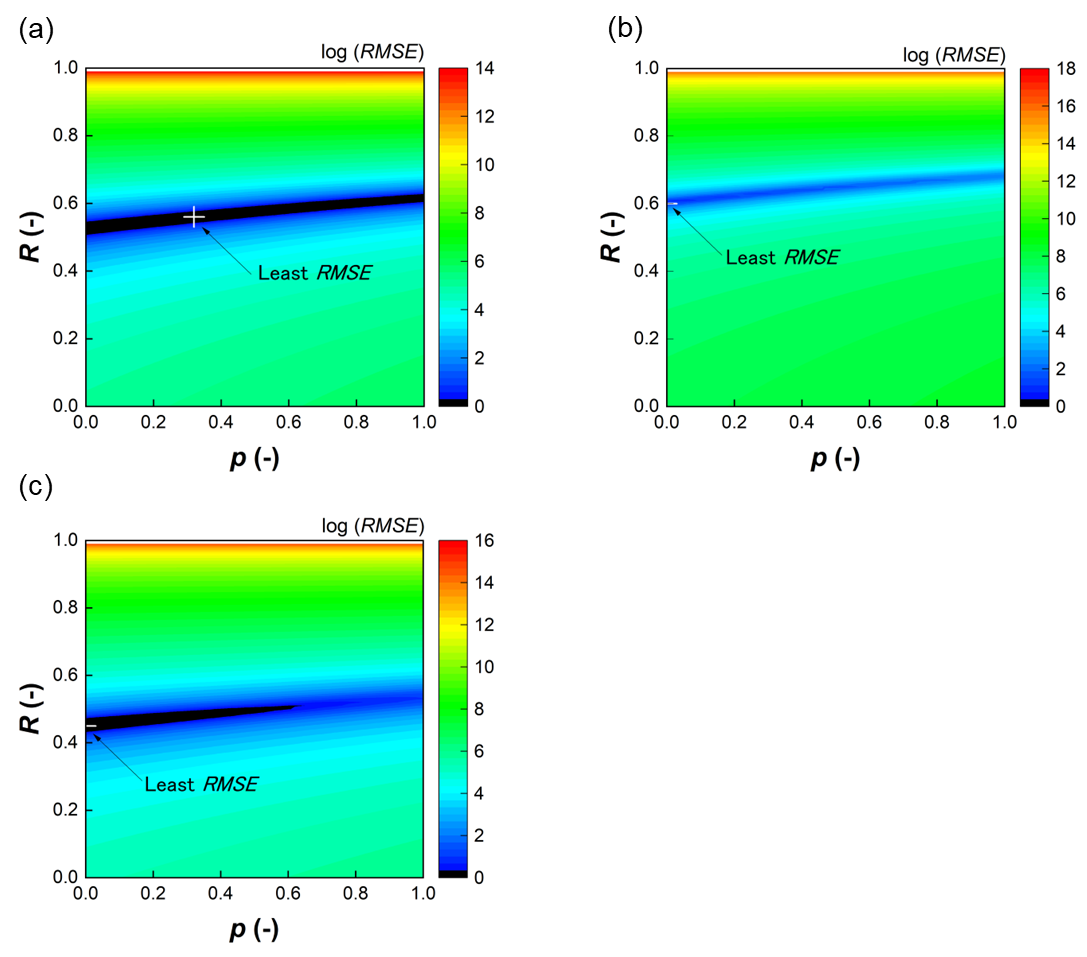


**Figure S19**. Root mean square error (RMSE) between experimental data and fitted curve to Fuchs-Sondheimer-Mayadas-Shatzkes model with logarithm scale (white cross marks are the least RMSE point) (A) 300 ^o^C deposition (B) 350 ^o^C deposition with < 25 nm (C) 350 ^o^C deposition with > 25 nm.


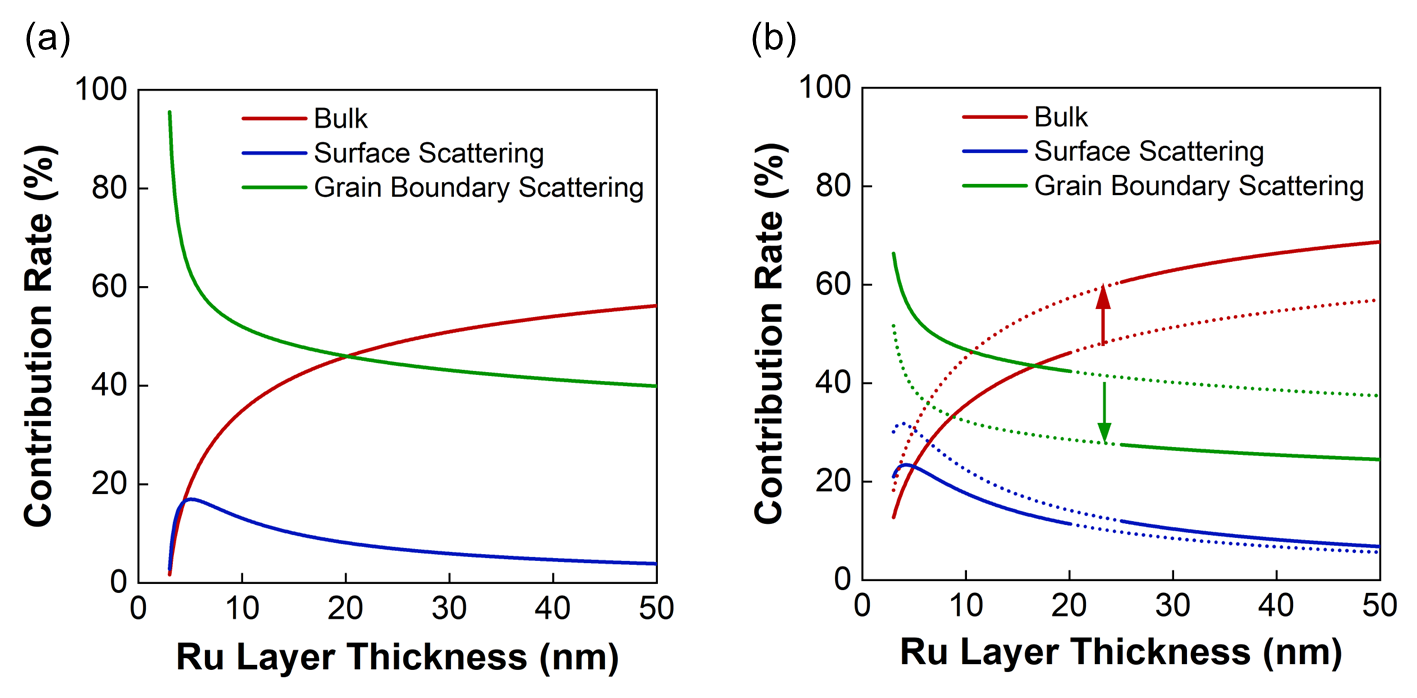


**Figure S20**. The contribution rate of bulk, surface scattering and grain boundary scattering to the resistivity of ALD-Ru thin films by adopting FS-MS model fitted with *ρ*_0_ = 7.4μΩ･cm, *λ* = 6.6 nm (A) at 300 ^o^C deposition, (B) at 350 ^o^C deposition.


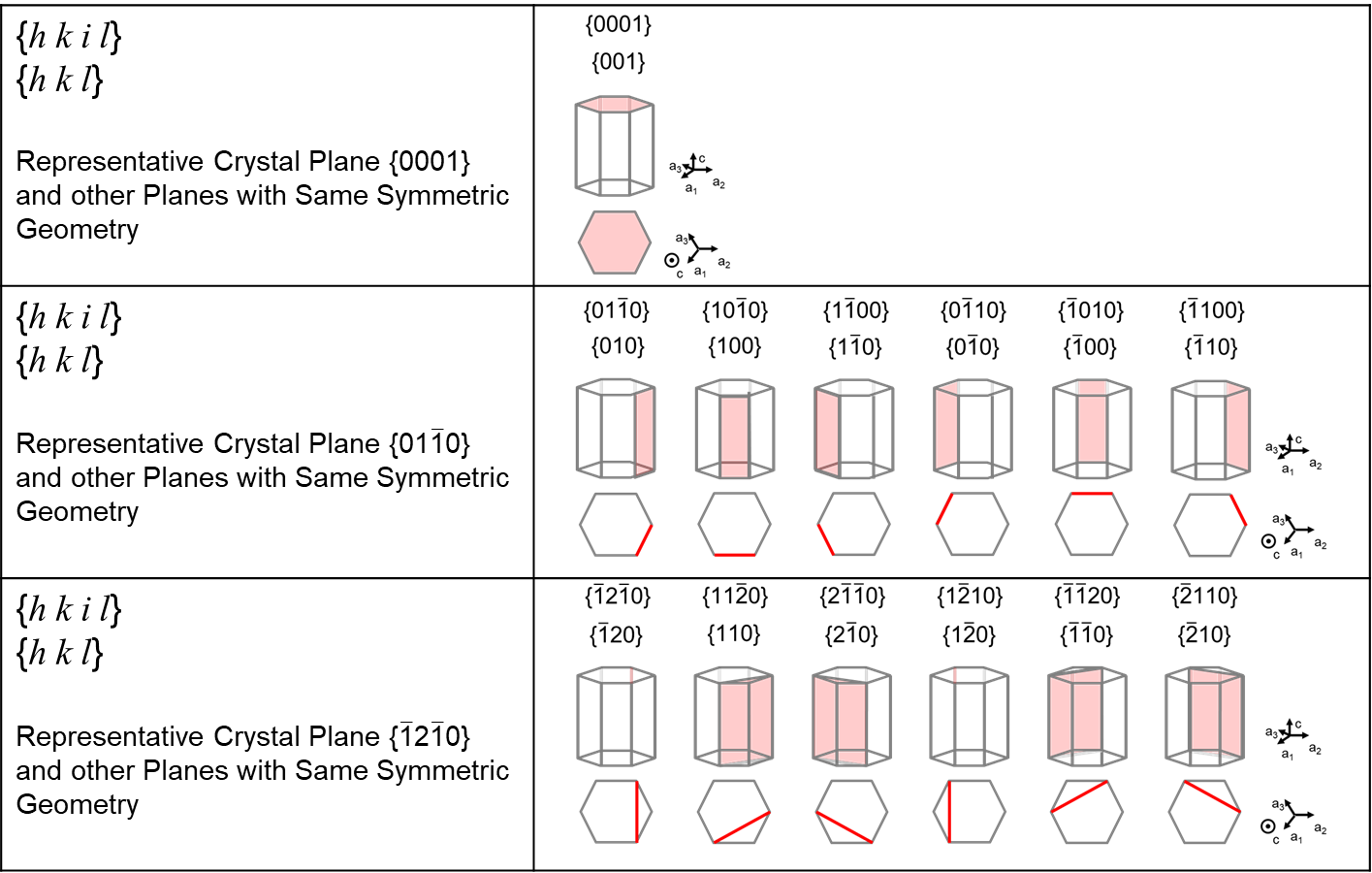


**Figure S21**. The representative crystal planes of the hcp structure and other planes have the same symmetric geometry.


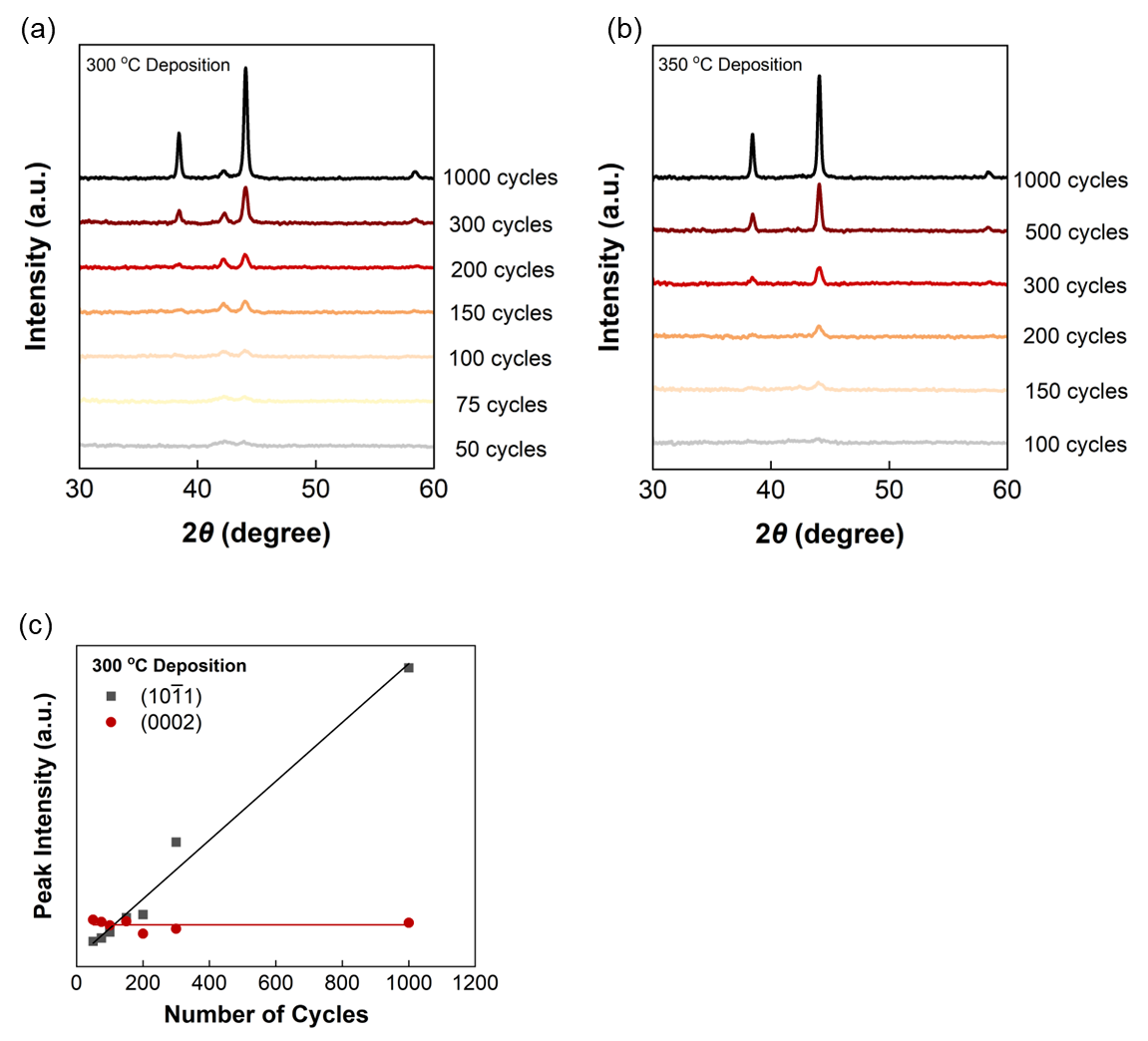


**Figure S22**. The diffraction peaks of Ru (10$\bar{1}$1) and (0002) with number of cycles at (A) 300 ^o^C deposition, (B) 350 ^o^C deposition, (C) peak intensities as a function of number of ALD cycles.


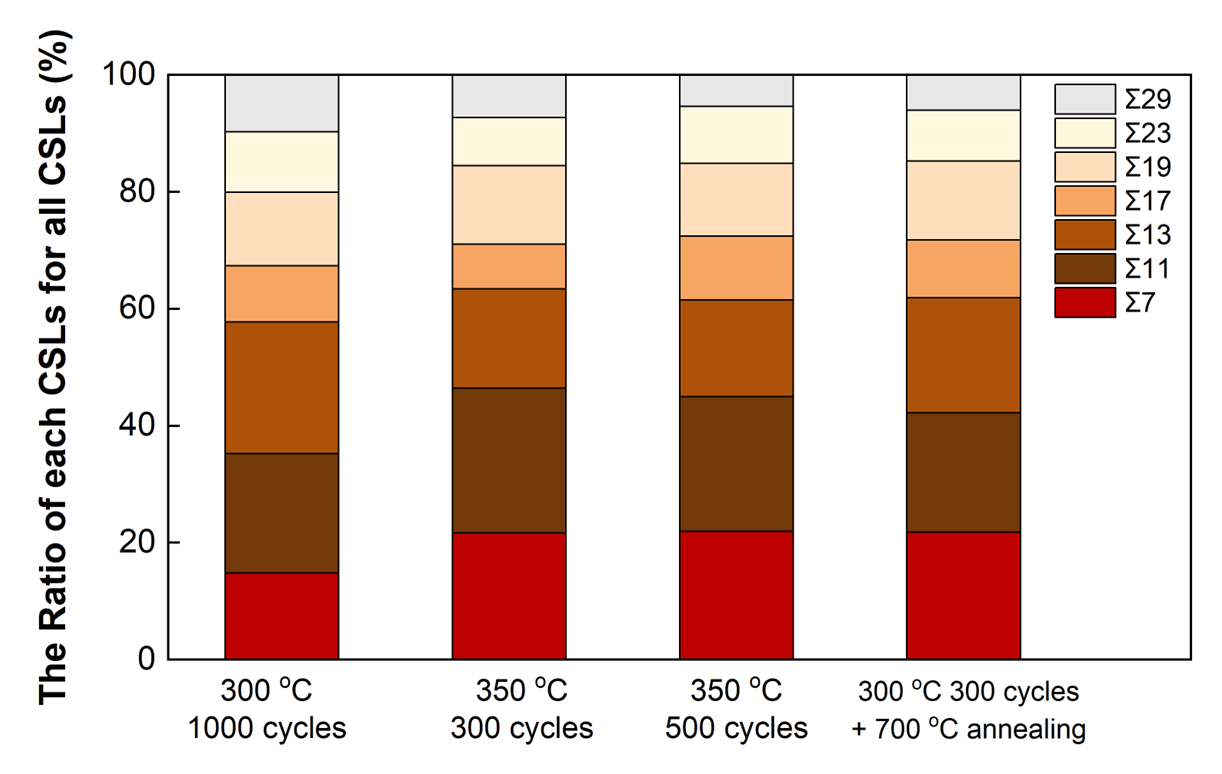


**Figure S23**. The ratio of each CSLs to all CSLs for the samples of 300 ^o^C 1000cycles, 350 ^o^C 300 cycles, 350 ^o^C 500 cycles and 300 ^o^C 300 cycles after 700 ^o^C annealing.

**Table S1.** Ru precursor properties and ALD-Ru thin films' physiochemical properties and character with different deposition methods

| Precursor | Metal oxidation state | Growth per cycle (GPC) [Å cycle^-1^] | Incubation cycles | Thermal decomposition temperature [^o^C] | Resistivity [μΩ cm] (temperature [^o^C], thickness [nm]) | References |
| --- | --- | --- | --- | --- | --- | --- |
| [Ru(thd)_3_]^a)^ | +3 | 0.36 | ~250 (Al_2_O_3_) | NA | 15-20 (NA, NA) | 34 |
| [Ru(DMPD)_2_]^b)^ | +2 | 0.12 | ~50 (SiO_2_) | >210 | NA | 35 |
| [Ru(chd)_2_]^c)^ | +2 | 0.2 | ~22 (SiO_2_), neglibigle (TiN) | NA | 14 (NA, 5.29) | 36 |
| Ru(C_7_H_7_O)(C_7_H_9_)^d)^ | +2 | 0.22 | ~30 (SiO_2_, TiN), negligible (Pt) | NA | 17-19 (200-250, 10-15) | 37 |
| DER^e)^ | +2 | 0.4 | negligible (SiO_2_, TiN) | NA | 19 (NA, ~10) | 38 |
| [Ru(Cp)_2_]^f)^ | +2 | 0.45 | ~250 (Al_2_O_3_) | NA | 13 (350, NA) | 39 |
| [Ru(EtCp)_2_]^g)^ | +2 | 0.49 | ~200 (SiO_2_) | NA | 15 (270, NA) | 40, 41 |
| Cyprus^h)^ | +2 | 0.5 | ~50 (SiO_2_) | NA | 20 (270, 25) | 42 |
| DMPR^i)^ | +2 | 0.55 | ~50 (SiO_2_) | NA | 18-24 (275, 9.7-16.6) | 43 |
| Rudense^j)^ | +2 | 0.83 | negligible (Pt), ~5 (Ta_2_O_5_) | NA | 23 (250, ~20) | 44 |
| ECPR^k)^ | +2 | 0.84 | ~20 (SiO_2_/Si) | 310 | 24 (255, 10.7) | 45 |
| CpRu(CO)_2_Et^l)^ | +2 | ~1 | ~100 (TiN) | 325 | 16 (~325, 25-30) | 46 |
| Carish^m)^ | +2 | ~1 | ~0 (SiO_2_/Si) | NA | 16-18 (247-283, NA) | 47 |
| EBECHRu^n)^ | 0 | 0.42 | 3 (SiO_2_) | NA | 20 (NA, NA) | 48 |
| EBBDRu^o)^ | 0 | ~0.56 | 15 (SiO_2_) | NA | ~26 (225, ~10) | 49 |
| [Ru(DMBD)(CO)_3_]^p)^ | 0 | 0.67 | ~0 (NA) | 300 | 14 (280, 8) | 50, 51 |
| (HD)iPrMePhRu^q)^ | 0 | 0.76 | 3 (SiO_2_) | NA | 29-36 (270-350, NA) | 52 |
| IMBCHDRu^r)^ | 0 | 0.89 | 11 (SiO_2_), ~0 (TiN) | 310 | ~30 (270, ~20) | 53, 54 |
| EBCHDRu^s)^ | 0 | 1 | 2 (SiO_2_) | 310 | ~20 (225, NA) | 55 |
| [Ru(TMM)(CO)_3_]^t)^ | +/0 | 1.7 | 6 (SiO_2_) | 275 | 12.9 (260, ~40), 20-25 (220, ~30) | 56 |
| **Ru(TMM)(p-cymene)^u)^** | **+/0** | **1.28** | **>1000 (SiO_2_), ~8 (TiN)** | **>400** | **11.0 (375, ~40), 14.0 (300, ~25)** | ***This work*** |

^a)^Tris(2,2,6,6-tetramethyl-3,5-heptanedionato)ruthenium(Ⅲ). ^b)^Bis(2,4-dimethylpentadienyl)ruthenium(Ⅱ). ^c)^Bis(*η*^5^-cycloheptadienyl)ruthenium(Ⅱ). ^d)^(*η*^5^-2,5-Cycloheptadien-1-one)-(*η*^5^-cycloheptadienyl)ruthenium(Ⅱ). ^e)^2,4-(Ethylcyclopentadienyl)-(dimethylpentadienyl)ruthenium(Ⅱ). ^f)^Bis(cyclopentadienyl)ruthenium(Ⅱ). ^g)^Bis(ethylcyclopentadienyl)ruthenium(Ⅱ). ^h)^Bis(2,6,6-trimethyl-cyclohexadienyl)ruthenium(Ⅱ). ^i)^Bis(2,5-dimethylpyrrolyl)ruthenium(Ⅱ). ^j)^(Ethylcyclopentadienyl)-bis(5-methyl-2,4-haxanediketonato)ruthenium(Ⅱ). ^k)^(Ethylcyclopentadienyl)-(pyrrolyl)ruthenium(Ⅱ). ^l)^Dicarbonyl(cyclopentadienyl)(ethyl)ruthenium(Ⅱ). ^m)^Dicarbonyl-bis(5-methyl-2,4-hexanediketonato)ruthenium(Ⅱ). ^n)^(Ethylbenzyl)(1-ethyl-1,4-cyclohaxadienyl)ruthenium(0). ^o)^(1,3-Butadiene)(ethylbenzene)ruthenium(0). ^p)^Tricarbonyl-(*η*^4^-2,3-dimethylbutadiene)ruthenium(0). ^q)^(1,5-Haxadiene)(1-isopropyl-4methylbenzene)ruthenium(0). ^r)^*η*^6^-1-Isopropyl-4-methylbenzene(*η*^4^-cyclohaxa-1,3-diene)ruthenium(0). ^s)^(1,3-Cyclohexadiene)(ethylbenzene)ruthenium(0). ^t)^Tricarbonyl(trimethylenemethane)ruthenium. ^u)^*η*^6^-1-Isopropyl-4-methylbenzene(trimethylenemethane)ruthenium.

**Table S2.** The amount of Ruthenium deposited quantified by X-ray fluorescence (XRF) (A) on SiO_2_ substrates (B) on TiN substrates.

(a)

| SiO_2_ | Bare | 75 cycles | 100 cycles | 150 cycles | 200 cycles | 300 cycles | 1000 cycles |
| --- | --- | --- | --- | --- | --- | --- | --- |
| Ru Lα signals [cps] | 1.161 | 0.562 | 0.741 | 0.000 | 1.166 | 0.000 | 1.413 |
| Ru layer thickness [nm] | 0.1 | 0.06 | 0.08 | 0.00 | 0.13 | 0.00 | 0.16 |

(b)

| TiN | 75 cycles | 100 cycles | 150 cycles | 200 cycles | 300 cycles | 1000 cycles |
| --- | --- | --- | --- | --- | --- | --- |
| Ru Lα signals [cps] | 101.9 | 127.9 | 200.9 | 280.5 | 353.2 | 898.6 |
| Ru layer thickness [nm] | 12.0 | 15.1 | 23.9 | 33.6 | 42.6 | 113.1 |

**Table S3.** MLIP-DFT comparison of representative bulk and surface properties for Ru and RuO₂ used in this study.

| Property | Material | MLIP | DFT |
| --- | --- | --- | --- |
| Cohesive Energy  [eV atom^-1^] | HCP Ru  (bulk) | 7.37 | 7.79 |
| Formation Energy  [eV f.u.^-1^] | Rutile RuO_2_  (bulk) | -3.34 | -3.17 |
| Surface Energy  [eV Å^-2^] | HCP Ru  (slab, (0001)) | 0.22 | 0.22 |
|  | Rutile RuO_2_  (slab, (110)) | 0.10 | 0.10 |
| Lattice Parameters  ; Lengths [Å], Angles [^o^ ] | HCP Ru  (bulk) | (a, b, c) = (2.71, 2.71, 4.27)  (α, β, γ) = (90, 90, 120) | (a, b, c) = (2.68, 2.68, 4.23)  (α, β, γ) = (90, 90, 120) |
|  | Rutile RuO_2_  (bulk) | (a, b, c) = (4.48, 4.48, 3.11)  (α, β, γ) = (90, 90, 90) | (a, b, c) = (4.48, 4.48, 3.11)  (α, β, γ) = (90, 90, 90) |

**Table S4.** Estimated surface adsorption lifetimes normalized to the attempt frequency (*τ*/*τ*₀), calculated from desorption energies using first-order Arrhenius kinetics at 300 °C.

| Ligand | Substrate | *t* /*t* _0_ at 300 ^o^C |
| --- | --- | --- |
| p-cymene | Ru | 9.1 ×10^19^ |
|  | RuO_2_ | 3.3 ×10^11^ |
|  | SiO_2_ | 5.2 ×10^5^ |
| TMM | Ru | 5.7 ×10^36^ |
|  | RuO_2_ | 2.0 ×10^34^ |
|  | SiO_2_ | 4.0 ×10^3^ |

**Table S5.** Supplementary BDE values for alternative ligand dissociation pathways in Ru(0) or Ru(+/0) precursors

| Precursor | Dissociation path | Homolytic BDE [eV] |
| --- | --- | --- |
| RuDMBD(CO)_3_ | Ru(CO)_3_+DMBD | 2.90 |
| Ru(TMM)(CO)_3_ | Ru(CO)_3_+TMM | 4.42 |
| IMBCHDRu | Ru(CHD) +Pcym | 3.08 |
|  | Ru(Pcym) +CHD | 4.46 |
| EBCHDRu | Ru(CHD)+EB | 3.07 |
|  | Ru(EB)+CHD | 4.42 |
| EBECHDRu | Ru(ECHD) +EB | 2.61 |
|  | Ru(EB) +ECHD | 3.78 |
| (HD)*i*PrMePhRu | Ru(Pcym)+HD | 4.53 |

**Table S6.** Rotation axis and angle of Coincidence site lattice (CSL) boundary of hcp structure with (c/a)^2^ = 2.5.

| # | Σ (Sigma) | *u v t w*  (Axis) | *θ*_d_ [°]  (Rotation Angle) |
| --- | --- | --- | --- |
| 1 | 7 | 0 0 0 1 | 21.8 |
| 2 | 7 | 1 0 -1 0 | 64.6 |
| 3 | 11 | 1 0 -1 0 | 35.1 |
| 4 | 11 | 2 -1 -1 0 | 84.8 |
| 5 | 13 | 0 0 0 1 | 27.8 |
| 6 | 13 | 2 -1 -1 0 | 57.4 |
| 7 | 13 | 1 0 -1 0 | 76.7 |
| 8 | 17 | 2 -1 -1 0 | 40.1 |
| 9 | 17 | 3 -1 -2 0 | 79.8 |
| 10 | 19 | 0 0 0 1 | 13.2 |
| 11 | 19 | 10 -5 -5 3 | 65.1 |
| 12 | 19 | 1 0 -1 0 | 87.0 |
| 13 | 23 | 1 0 -1 0 | 49.1 |
| 14 | 23 | 2 -1 -1 0 | 72.3 |
| 15 | 23 | 10 0 -10 3 | 86.3 |
| 16 | 29 | 2 -1 -1 0 | 49.1 |
| 17 | 29 | 20 10 -10 3 | 73.0 |
| 18 | 29 | 5 -1 -4 0 | 88.0 |

**References**

(1) Herberich, G. E.; Spaniol, T. P. Trimethylenemethane Complexes of Ruthenium, Osmium and Rhodium via the Compound CH_2_ C(CH_2_ SnMe_3_ )_2_. *J. Chem. Soc., Dalton Trans.* **1993**, No. 16, 2471–2476. https://doi.org/10.1039/DT9930002471.

(2) Bonnet, R.; Cousineau, E.; Warrington, D. H. Determination of Near-Coincident Cells for Hexagonal Crystals. Related DSC Lattices. *Acta Cryst A* **1981**, *37* (2), 184–189. https://doi.org/10.1107/S0567739481000466.

(3) Brandon, D. G. The Structure of High-Angle Grain Boundaries. *Acta Metallurgica* **1966**, *14* (11), 1479–1484. https://doi.org/10.1016/0001-6160(66)90168-4.

(4) Lin, H.; Liu, J.-X.; Fan, H.; Li, W.-X. Compensation between Surface Energy and Hcp/Fcc Phase Energy of Late Transition Metals from First-Principles Calculations. *J. Phys. Chem. C* **2020**, *124* (20), 11005–11014. https://doi.org/10.1021/acs.jpcc.0c02142.

(5) Xu, C.; Jiang, Y.; Yi, D.; Zhang, H.; Peng, S.; Liang, J. Prediction on the Surface Phase Diagram and Growth Morphology of Nanocrystal Ruthenium Dioxide. *Journal of the American Ceramic Society* **2014**, *97* (11), 3702–3709. https://doi.org/10.1111/jace.13148.

(6) Malyi, O. I.; Kulish, V. V.; Persson, C. In Search of New Reconstructions of (001) α-Quartz Surface: A First Principles Study. *RSC Adv.* **2014**, *4* (98), 55599–55603. https://doi.org/10.1039/C4RA10726H.

(7) Takamoto, S.; Shinagawa, C.; Motoki, D.; Nakago, K.; Li, W.; Kurata, I.; Watanabe, T.; Yayama, Y.; Iriguchi, H.; Asano, Y.; Onodera, T.; Ishii, T.; Kudo, T.; Ono, H.; Sawada, R.; Ishitani, R.; Ong, M.; Yamaguchi, T.; Kataoka, T.; Hayashi, A.; Charoenphakdee, N.; Ibuka, T. Towards Universal Neural Network Potential for Material Discovery Applicable to Arbitrary Combination of 45 Elements. *Nat Commun* **2022**, *13* (1), 2991. https://doi.org/10.1038/s41467-022-30687-9.

(8) Takamoto, S.; Okanohara, D.; Li, Q.-J.; Li, J. Towards Universal Neural Network Interatomic Potential. *Journal of Materiomics* **2023**, *9* (3), 447–454. https://doi.org/10.1016/j.jmat.2022.12.007.

(9) *MATLANTIS*. MATLANTIS. https://matlantis.com (accessed 2025-04-23).

(10) Kresse, G.; Furthmüller, J. Efficient Iterative Schemes for *Ab Initio* Total-Energy Calculations Using a Plane-Wave Basis Set. *Phys. Rev. B* **1996**, *54* (16), 11169–11186. https://doi.org/10.1103/PhysRevB.54.11169.

(11) Perdew, J. P.; Burke, K.; Ernzerhof, M. Generalized Gradient Approximation Made Simple. *Phys. Rev. Lett.* **1996**, *77* (18), 3865–3868. https://doi.org/10.1103/PhysRevLett.77.3865.

(12) Grimme, S.; Antony, J.; Ehrlich, S.; Krieg, H. A Consistent and Accurate *Ab Initio* Parametrization of Density Functional Dispersion Correction (DFT-D) for the 94 Elements H-Pu. *The Journal of Chemical Physics* **2010**, *132* (15), 154104. https://doi.org/10.1063/1.3382344.

(13) Grimme, S.; Ehrlich, S.; Goerigk, L. Effect of the Damping Function in Dispersion Corrected Density Functional Theory. *J Comput Chem* **2011**, *32* (7), 1456–1465. https://doi.org/10.1002/jcc.21759.

(14) Paszke, A.; Gross, S.; Massa, F.; Lerer, A.; Bradbury, J.; Chanan, G.; Killeen, T.; Lin, Z.; Gimelshein, N.; Antiga, L.; Desmaison, A.; Kopf, A.; Yang, E.; DeVito, Z.; Raison, M.; Tejani, A.; Chilamkurthy, S.; Steiner, B.; Fang, L.; Bai, J.; Chintala, S. PyTorch: An Imperative Style, High-Performance Deep Learning Library.

(15) Hjorth Larsen, A.; Jørgen Mortensen, J.; Blomqvist, J.; Castelli, I. E.; Christensen, R.; Dułak, M.; Friis, J.; Groves, M. N.; Hammer, B.; Hargus, C.; Hermes, E. D.; Jennings, P. C.; Bjerre Jensen, P.; Kermode, J.; Kitchin, J. R.; Leonhard Kolsbjerg, E.; Kubal, J.; Kaasbjerg, K.; Lysgaard, S.; Bergmann Maronsson, J.; Maxson, T.; Olsen, T.; Pastewka, L.; Peterson, A.; Rostgaard, C.; Schiøtz, J.; Schütt, O.; Strange, M.; Thygesen, K. S.; Vegge, T.; Vilhelmsen, L.; Walter, M.; Zeng, Z.; Jacobsen, K. W. The Atomic Simulation Environment—a Python Library for Working with Atoms. *J. Phys.: Condens. Matter* **2017**, *29* (27), 273002. https://doi.org/10.1088/1361-648X/aa680e.

(16) Neese, F. Software Update: The ORCA Program System—Version 5.0. *WIREs Computational Molecular Science* **2022**, *12* (5), e1606. https://doi.org/10.1002/wcms.1606.

(17) Adamo, C.; Barone, V. Toward Reliable Density Functional Methods without Adjustable Parameters: The PBE0 Model. *The Journal of Chemical Physics* **1999**, *110* (13), 6158–6170. https://doi.org/10.1063/1.478522.

(18) Grimme, S.; Ehrlich, S.; Goerigk, L. Effect of the Damping Function in Dispersion Corrected Density Functional Theory. *J Comput Chem* **2011**, *32* (7), 1456–1465. https://doi.org/10.1002/jcc.21759.

(19) Grimme, S. Supramolecular Binding Thermodynamics by Dispersion-Corrected Density Functional Theory. *Chemistry – A European Journal* **2012**, *18* (32), 9955–9964. https://doi.org/10.1002/chem.201200497.

(20) Weigend, F.; Ahlrichs, R. Balanced Basis Sets of Split Valence, Triple Zeta Valence and Quadruple Zeta Valence Quality for H to Rn: Design and Assessment of Accuracy. *Phys. Chem. Chem. Phys.* **2005**, *7* (18), 3297. https://doi.org/10.1039/b508541a.

(21) Gall, D. The Search for the Most Conductive Metal for Narrow Interconnect Lines. *Journal of Applied Physics* **2020**, *127* (5), 050901. https://doi.org/10.1063/1.5133671.

(22) Ke, Y.; Zahid, F.; Timoshevskii, V.; Xia, K.; Gall, D.; Guo, H. Resistivity of Thin Cu Films with Surface Roughness. *Phys. Rev. B* **2009**, *79* (15), 155406. https://doi.org/10.1103/PhysRevB.79.155406.

(23) Zhou, T.; Zheng, P.; Pandey, S. C.; Sundararaman, R.; Gall, D. The Electrical Resistivity of Rough Thin Films: A Model Based on Electron Reflection at Discrete Step Edges. *Journal of Applied Physics* **2018**, *123* (15), 155107. https://doi.org/10.1063/1.5020577.

(24) Zheng, P. Y.; Zhou, T.; Engler, B. J.; Chawla, J. S.; Hull, R.; Gall, D. Surface Roughness Dependence of the Electrical Resistivity of W(001) Layers. *Journal of Applied Physics* **2017**, *122* (9), 095304. https://doi.org/10.1063/1.4994001.

(25) Milosevic, E.; Kerdsongpanya, S.; Zangiabadi, A.; Barmak, K.; Coffey, K. R.; Gall, D. Resistivity Size Effect in Epitaxial Ru(0001) Layers. *Journal of Applied Physics* **2018**, *124* (16), 165105. https://doi.org/10.1063/1.5046430.

(26) Kim, Y.-H.; Kotsugi, Y.; Cheon, T.; Ramesh, R.; Kim, S.-H. Atomic Layer Deposition of RuO _2_ Using a New Metalorganic Precursor as a Diffusion Barrier for Ru Interconnect. In *2021 IEEE International Interconnect Technology Conference (IITC)*; IEEE: Kyoto, Japan, 2021; pp 1–3. https://doi.org/10.1109/IITC51362.2021.9537498.

(27) Lehto, P. Adaptive Domain Misorientation Approach for the EBSD Measurement of Deformation Induced Dislocation Sub-Structures. *Ultramicroscopy* **2021**, *222*, 113203. https://doi.org/10.1016/j.ultramic.2021.113203.

(28) Markov, I. V. *Crystal Growth for Beginners: Fundamentals of Nucleation, Crystal Growth, and Epitaxy*, 3rd edition.; World Scientific: New Jersey London Singapore Beijing Shanghai Hong Kong Taipei Chennai Tokyo, 2017. https://doi.org/10.1142/10127.

(29) Kobayashi, S.; Takagi, H.; Watanabe, T. Grain Boundary Character Distribution and Texture Evolution during Surface Energy-Driven Grain Growth in Nanocrystalline Gold Thin Films. *Philosophical Magazine* **2013**, *93* (10–12), 1425–1442. https://doi.org/10.1080/14786435.2012.756991.

(30) Priester, L. *Grain Boundaries: From Theory to Engineering*; Springer Series in Materials Science; Springer Netherlands: Dordrecht, 2013; Vol. 172. https://doi.org/10.1007/978-94-007-4969-6.

(31) Wulff, G. On the Question of Speed of Growth and Dissolution of Crystal Surfaces. *Z. Kristallogr* **1901**, *34* (5/6), 449.

(32) Gottstein, G. N.; Molodov, D. A.; Shvindlerman, L. S. Grain Boundary Migration in Metals: Recent Developments. **1998**.

(33) Watanabe, T. Grain Boundary Engineering: Historical Perspective and Future Prospects. *J Mater Sci* **2011**, *46* (12), 4095–4115. https://doi.org/10.1007/s10853-011-5393-z.

(34) Aaltonen, T.; Ritala, M.; Arstila, K.; Keinonen, J.; Leskelä, M. Atomic Layer Deposition of Ruthenium Thin Films from Ru(Thd) _3_ and Oxygen. *Chemical Vapor Deposition* **2004**, *10* (4), 215–219. https://doi.org/10.1002/cvde.200306288.

(35) Methaapanon, R.; Geyer, S. M.; Lee, H.-B.-R.; Bent, S. F. The Low Temperature Atomic Layer Deposition of Ruthenium and the Effect of Oxygen Exposure. *J. Mater. Chem.* **2012**, *22* (48), 25154. https://doi.org/10.1039/c2jm35332f.

(36) Hwang, J. M.; Han, S.-M.; Yang, H.; Yeo, S.; Lee, S.-H.; Park, C. W.; Kim, G. H.; Park, B. K.; Byun, Y.; Eom, T.; Chung, T.-M. Atomic Layer Deposition of a Ruthenium Thin Film Using a Precursor with Enhanced Reactivity. *J. Mater. Chem. C* **2021**, *9* (11), 3820–3825. https://doi.org/10.1039/D0TC05682K.

(37) Oh, S. H.; Hwang, J. M.; Park, H.; Park, D.; Song, Y. E.; Ko, E. C.; Park, T. J.; Eom, T.; Chung, T. Atomic Layer Deposition of Ru Thin Film Using a Newly Synthesized Precursor with Open‐Coordinated Ligands. *Adv Materials Inter* **2023**, *10* (17), 2202445. https://doi.org/10.1002/admi.202202445.

(38) Kim, S. K.; Lee, S. Y.; Lee, S. W.; Hwang, G. W.; Hwang, C. S.; Lee, J. W.; Jeong, J. Atomic Layer Deposition of Ru Thin Films Using 2,4-(Dimethylpentadienyl)(Ethylcyclopentadienyl)Ru by a Liquid Injection System. *Journal of The Electrochemical Society*.

(39) Aaltonen, T.; Alén, P.; Ritala, M.; Leskelä, M. Ruthenium Thin Films Grown by Atomic Layer Deposition. *Chemical Vapor Deposition* **2003**, *9* (1), 45–49. https://doi.org/10.1002/cvde.200290007.

(40) Kwon, O.-K.; Kim, J.-H.; Park, H.-S.; Kang, S.-W. Atomic Layer Deposition of Ruthenium Thin Films for Copper Glue Layer. *J. Electrochem. Soc.* **2004**, *151* (2), G109. https://doi.org/10.1149/1.1640633.

(41) Yim, S.-S.; Lee, D.-J.; Kim, K.-S.; Kim, S.-H.; Yoon, T.-S.; Kim, K.-B. Nucleation Kinetics of Ru on Silicon Oxide and Silicon Nitride Surfaces Deposited by Atomic Layer Deposition. *Journal of Applied Physics* **2008**, *103* (11), 113509. https://doi.org/10.1063/1.2938052.

(42) Gregorczyk, K.; Henn-Lecordier, L.; Gatineau, J.; Dussarrat, C.; Rubloff, G. Atomic Layer Deposition of Ruthenium Using the Novel Precursor Bis(2,6,6-Trimethyl-Cyclohexadienyl)Ruthenium. *Chem. Mater.* **2011**, *23* (10), 2650–2656. https://doi.org/10.1021/cm2004825.

(43) Kukli, K.; Aarik, J.; Aidla, A.; Jõgi, I.; Arroval, T.; Lu, J.; Sajavaara, T.; Laitinen, M.; Kiisler, A.-A.; Ritala, M.; Leskelä, M.; Peck, J.; Natwora, J.; Geary, J.; Spohn, R.; Meiere, S.; Thompson, D. M. Atomic Layer Deposition of Ru Films from Bis(2,5-Dimethylpyrrolyl)Ruthenium and Oxygen. *Thin Solid Films* **2012**, *520* (7), 2756–2763. https://doi.org/10.1016/j.tsf.2011.11.088.

(44) Kwon, D. S.; An, C. H.; Kim, S. H.; Kim, D. G.; Lim, J.; Jeon, W.; Hwang, C. S. Atomic Layer Deposition of Ru Thin Films Using (2,4-Dimethyloxopentadienyl)(Ethylcyclopentadienyl)Ru and the Effect of Ammonia Treatment during the Deposition. *J. Mater. Chem. C* **2020**, *8* (21), 6993–7004. https://doi.org/10.1039/D0TC01489C.

(45) Knaut, M.; Junige, M.; Albert, M.; Bartha, J. W. *In-Situ* Real-Time Ellipsometric Investigations during the Atomic Layer Deposition of Ruthenium: A Process Development from [(Ethylcyclopentadienyl)(Pyrrolyl)Ruthenium] and Molecular Oxygen. *Journal of Vacuum Science & Technology A: Vacuum, Surfaces, and Films* **2012**, *30* (1), 01A151. https://doi.org/10.1116/1.3670405.

(46) Leick, N.; Verkuijlen, R. O. F.; Lamagna, L.; Langereis, E.; Rushworth, S.; Roozeboom, F.; Van De Sanden, M. C. M.; Kessels, W. M. M. Atomic Layer Deposition of Ru from CpRu(CO)2Et Using O2 Gas and O2 Plasma. *Journal of Vacuum Science & Technology A: Vacuum, Surfaces, and Films* **2011**, *29* (2), 021016. https://doi.org/10.1116/1.3554691.

(47) Nguyen, C. T.; Yoon, J.; Khan, R.; Shong, B.; Lee, H.-B.-R. Thermal Atomic Layer Deposition of Metallic Ru Using H2O as a Reactant. *Applied Surface Science* **2019**, *488*, 896–902. https://doi.org/10.1016/j.apsusc.2019.05.242.

(48) Popovici, M.; Groven, B.; Marcoen, K.; Phung, Q. M.; Dutta, S.; Swerts, J.; Meersschaut, J.; Van Den Berg, J. A.; Franquet, A.; Moussa, A.; Vanstreels, K.; Lagrain, P.; Bender, H.; Jurczak, M.; Van Elshocht, S.; Delabie, A.; Adelmann, C. Atomic Layer Deposition of Ruthenium Thin Films from (Ethylbenzyl) (1-Ethyl-1,4-Cyclohexadienyl) Ru: Process Characteristics, Surface Chemistry, and Film Properties. *Chem. Mater.* **2017**, *29* (11), 4654–4666. https://doi.org/10.1021/acs.chemmater.6b05437.

(49) Yeo, S.; Park, J.-Y.; Lee, S.-J.; Lee, D.-J.; Seo, J. H.; Kim, S.-H. Ruthenium and Ruthenium Dioxide Thin Films Deposited by Atomic Layer Deposition Using a Novel Zero-Valent Metalorganic Precursor, (Ethylbenzene)(1,3-Butadiene)Ru(0), and Molecular Oxygen. *Microelectronic Engineering* **2015**, *137*, 16–22. https://doi.org/10.1016/j.mee.2015.02.026.

(50) Cwik, S.; Woods, K. N.; Saly, M. J.; Knisley, T. J.; Winter, C. H. Thermal Atomic Layer Deposition of Ruthenium Metal Thin Films Using Nonoxidative Coreactants. *Journal of Vacuum Science & Technology A: Vacuum, Surfaces, and Films* **2020**, *38* (1), 012402. https://doi.org/10.1116/1.5125109.

(51) Austin, D. Z.; Jenkins, M. A.; Allman, D.; Hose, S.; Price, D.; Dezelah, C. L.; Conley, J. F. Correction to Atomic Layer Deposition of Ruthenium and Ruthenium Oxide Using a Zero Oxidation State Precursor. *Chem. Mater.* **2018**, *30* (24), 8983–8984. https://doi.org/10.1021/acs.chemmater.8b04816.

(52) Jung, H. J.; Han, J. H.; Jung, E. A.; Park, B. K.; Hwang, J.-H.; Son, S. U.; Kim, C. G.; Chung, T.-M.; An, K.-S. Atomic Layer Deposition of Ruthenium and Ruthenium Oxide Thin Films from a Zero-Valent (1,5-Hexadiene)(1-Isopropyl-4-Methylbenzene)Ruthenium Complex and O_2_. *Chem. Mater.* **2014**, *26* (24), 7083–7090. https://doi.org/10.1021/cm5035485.

(53) Choi, S.-H.; Cheon, T.; Kim, S.-H.; Kang, D.-H.; Park, G.-S.; Kim, S. Thermal Atomic Layer Deposition (ALD) of Ru Films for Cu Direct Plating. *J. Electrochem. Soc.* **2011**, *158* (6), D351. https://doi.org/10.1149/1.3575163.

(54) Eom, T.-K.; Sari, W.; Choi, K.-J.; Shin, W.-C.; Kim, J. H.; Lee, D.-J.; Kim, K.-B.; Sohn, H.; Kim, S.-H. Low Temperature Atomic Layer Deposition of Ruthenium Thin Films Using Isopropylmethylbenzene-Cyclohexadiene-Ruthenium and O2.

(55) Yeo, S.; Choi, S.-H.; Park, J.-Y.; Kim, S.-H.; Cheon, T.; Lim, B.-Y.; Kim, S. Atomic Layer Deposition of Ruthenium (Ru) Thin Films Using Ethylbenzen-Cyclohexadiene Ru(0) as a Seed Layer for Copper Metallization. *Thin Solid Films* **2013**, *546*, 2–8. https://doi.org/10.1016/j.tsf.2013.03.074.

(56) Kotsugi, Y.; Han, S.-M.; Kim, Y.-H.; Cheon, T.; Nandi, D. K.; Ramesh, R.; Yu, N.-K.; Son, K.; Tsugawa, T.; Ohtake, S.; Harada, R.; Park, Y.-B.; Shong, B.; Kim, S.-H. Atomic Layer Deposition of Ru for Replacing Cu-Interconnects. *Chem. Mater.* **2021**, *33* (14), 5639–5651. https://doi.org/10.1021/acs.chemmater.1c01054.
